# The Protein Kinase Tor1 Regulates Adhesin Gene Expression in *Candida albicans*

**DOI:** 10.1371/journal.ppat.1000294

**Published:** 2009-02-06

**Authors:** Robert J. Bastidas, Joseph Heitman, Maria E. Cardenas

**Affiliations:** 1 Department of Molecular Genetics and Microbiology, Duke University Medical Center, Durham, North Carolina, United States of America; 2 Department of Medicine, Duke University Medical Center, Durham, North Carolina, United States of America; 3 Department of Pharmacology and Cancer Biology, Duke University Medical Center, Durham, North Carolina, United States of America; David Geffen School of Medicine at University of California Los Angeles, United States of America

## Abstract

Eukaryotic cell growth is coordinated in response to nutrient availability, growth factors, and environmental stimuli, enabling cell–cell interactions that promote survival. The rapamycin-sensitive Tor1 protein kinase, which is conserved from yeasts to humans, participates in a signaling pathway central to cellular nutrient responses. To gain insight into Tor-mediated processes in human fungal pathogens, we have characterized Tor signaling in *Candida albicans*. Global transcriptional profiling revealed evolutionarily conserved roles for Tor1 in regulating the expression of genes involved in nitrogen starvation responses and ribosome biogenesis. Interestingly, we found that in *C. albicans* Tor1 plays a novel role in regulating the expression of several cell wall and hyphal specific genes, including adhesins and their transcriptional repressors Nrg1 and Tup1. In accord with this transcriptional profile, rapamycin induced extensive cellular aggregation in an adhesin-dependent fashion. Moreover, adhesin gene induction and cellular aggregation of rapamycin-treated cells were strongly dependent on the transactivators Bcr1 and Efg1. These findings support models in which Tor1 negatively controls cellular adhesion by governing the activities of Bcr1 and Efg1. Taken together, these results provide evidence that Tor1-mediated cellular adhesion might be broadly conserved among eukaryotic organisms.

## Author Summary

Living organisms have an intrinsic ability to coordinate their growth and proliferation in response to nutrient availability. In organisms ranging from yeasts to humans, the Tor1 signaling pathway responds to nutrient-derived signals and orchestrates cell growth. Accordingly, we find that in the human fungal pathogen *Candida albicans*, Tor1 signaling also functions to promote growth. We also uncovered a novel role for the Tor1 molecular pathway in promoting hyphal growth of *C. albicans* on semi-solid surfaces and in controlling cell–cell adherence. Gene expression analysis and genetic manipulations implicate the known cell surface adhesins Als1 and Als3 as mediators of Tor1-regulated cellular adhesion. Further genetic analysis identified the transcriptional regulators Bcr1, Efg1, Nrg1, and Tup1 that together with Tor1 compose a regulatory network governing adhesin gene expression and cellular adhesion. Given that the Tor pathway is the target of several small molecule inhibitors including rapamycin, a versatile pharmacological drug used in medicine, there is considerable interest in Tor signaling pathways and their function. Moreover, given the potent fungicidal activity of rapamycin against *C. albicans*, novel antifungal therapies remain to be developed, which may also include novel antifungal therapies with less immunosuppressive rapamycin analogs.

## Introduction

Coordinated cell–cell adhesion is an essential biological process widely employed by organisms throughout the tree of life. In metazoans, cellular adhesion is important for numerous processes ranging from establishment of body plans and maintenance of differentiated tissues to regulation of cancer progression (reviewed in [1],[2]). Bacterial and fungal species commonly rely on cellular adhesion during mating and conjugation and for maintenance of multicellular biofilms that function as anchored shields against foreign attack by antimicrobial agents. In essence, cellular adhesion has driven important evolutionary benefits across kingdoms, ranging from the evolution of multicellularity in metazoans to drug resistance in bacteria and fungi.

Adhesion plays major roles in virulence-associated traits of several fungal pathogens. In *Candida albicans*, the most pervasive human fungal pathogen, cellular adhesion is essential for biofilm development. *C. albicans* biofilms form on both biotic and abiotic surfaces (such as tissues, plastic prosthesis, dentures and catheters) [3] and function as reservoirs of infective cells that among immunocompromised individuals can cause deep seated and often fatal mycosis [4]. The typical architecture of a biofilm consists of mixed layers of intertwined yeast and hyphal cells stabilized by adhesive interactions among neighboring cells. These cell–cell adhesive interactions are mediated by a set of cell surface displayed adhesins, including the Als proteins and the cell wall protein Hwp1 [5],[6],[7],[8]. The *ALS* genes *ALS1* and *ALS3*, two of eight *ALS* family members, are required for adherent interactions during biofilm formation in both *in-vitro* and *in-vivo* models of catheter biofilm formation and appear to have redundant functions [6]. *HWP1*, which codes for a cell surface glycoprotein targeted by mammalian transglutaminase that links Hwp1 to proteins on the mammalian cell surface (reviewed in [9]), surprisingly is also required for cell adhesive interactions during biofilm formation [6],[7]. Notably, Als1, Als3 and Hwp1 play complementary roles during biofilm formation suggesting that they might interact to promote adhesion between adjacent cellular surfaces [8].

Adhesin regulation in *C. albicans* occurs primarily at the transcriptional level. During biofilm formation, expression of *ALS1*, *ALS3* and *HWP1* is regulated by the transcription factor Bcr1 [5]. Additional factors, such as the transcription factors Tec1, and the repressors Nrg1 and Tup1, have also been implicated in regulating adhesin expression [10],[11],[12],[13],[14],[15],[16]. Furthermore, both *ALS3* and *HWP1* are developmentally regulated and exclusively expressed in *C. albicans* hyphae [17],[18]. This level of regulation falls under the domain of the cAMP-protein kinase-A signaling pathway that regulates yeast-hypha morphogenesis via the transcription factor Efg1 in response to nutritional and environmental cues [19],[20]. However, aside from the cAMP signaling pathway, little is known about additional molecular pathways that transduce nutritional signals to the multiple transcriptional regulators governing adhesin expression.

Our understanding of signaling networks regulating virulence traits in *C. albicans* in response to nutritional cues has relied heavily on previous knowledge of homologous pathways in the model yeast *Saccharomyces cerevisiae*. Nevertheless, despite the high similarity in gene content between both species, several lines of evidence now suggest that rewiring of regulatory networks is an increasingly common paradigm in *C. albicans* [21],[22],[23]. Following these lines of evidence, we sought to assess whether rewiring has also occurred in the nutrient responsive signal transduction pathway centered on the globally conserved protein kinase Tor.

Tor protein kinases were first identified in *S. cerevisiae* as the target of the antifungal and immunosuppressive agent rapamycin, which inhibits Tor function as an FKBP12-rapamycin complex [24]. Two Tor proteins, Tor1 and Tor2, have been characterized in *S. cerevisiae* and *Schizosaccharomyces pombe*, whereas a single Tor homolog is present in *C. albicans*, *Cryptococcus neoformans*, *Drosophila melanogaster*, and humans [24],[25],[26],[27],[28],[29],[30],[31]. Both of the *C. albicans* and *C. neoformans* Tor homologs are inhibited by conserved FKBP12-rapamycin mechanisms [27],[28]. In eukaryotic organisms ranging from yeast to humans, the Tor signaling pathway functions as a global regulator of cellular growth in response to nutritional cues (reviewed in [32]). In *S. cerevisiae*, inhibition of Tor signaling by FKBP12-rapamycin triggers autophagy, inhibits translation, represses ribosomal gene expression and induces expression of retrograde response (RTG), nitrogen catabolite regulated (NCR) and stress responsive (STRE) genes (reviewed in [33]).

In this study, we have characterized the transcriptional programs regulated by *C. albicans* Tor1 and demonstrate an evolutionarily conserved paradigm for Tor1 signaling in regulating transcriptional responses to nutrient starvation in both *C. albicans* and *S. cerevisiae*. Interestingly, our analysis has also uncovered a novel role for Tor1 signaling in regulating cell–cell adhesion in *C. albicans*. Tor1 activity represses adhesin gene expression and inhibition of Tor1 promotes cell–cell adhesion, a process that is central to biofilm development. Furthermore, through genetic and molecular approaches we have identified Tor1 as a constituent of the network involving the transcriptional regulators Bcr1, Efg1, Nrg1, and Tup1 that governs adhesin expression.

In summary our results forge a link between the nutrient-responsive Tor signal transduction cascade and regulation of cell–cell adhesion in a major human fungal pathogen and suggest that this function could be conserved in more complex organisms, including metazoans.

## Results

### Tor1 regulates hyphal-specific genes and transcriptional programs conserved with *S. cerevisiae*

The goal of this study was to characterize and define Tor1-dependent transcriptional responses in *C. albicans*. Tor1 is essential in *C. albicans* (our unpublished results) precluding the use of mutant strains lacking Tor1. To overcome this limitation, we compared the genome-wide transcriptional profile of wild type yeast cells grown in YPD liquid medium at 30°C exposed or not exposed to sublethal concentrations (20 nM) of the Tor1 specific inhibitor rapamycin. Gene expression analysis indicated that the abundance of over 400 transcripts changed upon rapamycin treatment (Table S1). Among these, 330 transcripts showed a greater than 2-fold reduction in expression levels. Strikingly, the expression of a large cluster of genes (∼120) encoding components of the translational machinery was downregulated in response to rapamycin treatment (Table S1). This includes genes encoding cytoplasmic ribosomal proteins, rRNA processing enzymes, several RNA polymerase III subunits, translation initiation and elongation factors, and tRNA synthetases. In addition, expression of mitochondrial ribosomal protein and amino acid biosynthetic genes was also repressed (Table S2). These results are consistent with previous reports showing coordinate repression of the ribosome biogenesis (Ribi) regulon during rapamycin treatment of *S. cerevisiae* cells [34],[35],[36],[37], and indicates that the role of Tor1 in translation regulation is evolutionarily conserved in *C. albicans*.

Exposure to rapamycin also resulted in pronounced induction of nitrogen catabolite repressed (NCR) genes such as those encoding the permeases Gap2, Mep2, Can1 and Can2 and the transporters Hip1, Dip5 and Dur3 (Table S2). Several other NCR genes were concurrently induced, including genes coding for allantoicase (Dal2), arginase (Car1), glutamate dehydrogenase (Gdh2), and the transcriptional regulators Ure2 and Gat1 (Table S2). Expression of *GLN3*, which encodes a well-characterized transactivator of the NCR response, did not show significant changes, which could be due to experimental conditions or posttranscriptional regulation of Gln3 by Tor1 signaling. Nevertheless, the coordinate expression of this cluster of genes strongly implicates Tor1 as a regulator of the NCR response in *C. albicans*, and mirrors the same pattern of expression found during Tor inhibition in *S. cerevisiae* cells [35],[36],[38],[39].

In addition to inducing NCR gene expression, rapamycin treatment resulted in increased gene expression of the amino acid starvation transcriptional regulator Gcn4, along with several carboxypeptidases, aminopeptidases, and oligopeptide transporter genes (Table S2). This transcriptional pattern indicates that rapamycin treated cells perceive the environment as nutrient limiting.

Interestingly, our analysis also revealed a unique role for Tor1 signaling in regulating a set of *C. albicans*-specific transcripts. Rapamycin treatment resulted in strong induction of classic hyphae-induced genes such as those encoding the adhesins Als1 and Als3, the cell wall proteins Rbt1 and Sun41, and the hyphae-specific protein Ece1 (Table 1). Furthermore, expression of the transcriptional regulators Rfg1, Czf1 and Tec1, which control hyphal-specific gene transcription required for filamentous growth, was also upregulated (Table 1).

**Table 1:** Rapamycin induction of hyphae-specific and cell wall transcripts.

Functional Category	orf19 Id	Locus Name	*S. cerevisiae* Best Hit	Fold Change	Description
**Hyphae-induced**	orf19.5741	*ALS1*	*FLO9*	29.7	Adhesin
	orf19.1327	*RBT1*	*MUC1*	7.2	Predicted cell wall protein
	orf19.2355	*ALS3*	*FLO1*	7.0	Adhesin
	orf19.3374	*ECE1*	*—*	6.1	Protein of unknown function
	orf19.3642	*SUN41*	*SIM1*	4.1	Putative cell wall protein
**Transcriptional regulation**	orf19.2823	*RFG1*	*ROX1*	6.2	Transcriptional regulator
	orf19.3127	*CZF1*	*UME6*	5.9	Transcriptional regulator
	orf19.5908	*TEC1*	*TEC1*	4.2	Transcriptional regulator
**Cell wall proteins**	orf19.1097	*ALS2*	*FLO1*	9.2	Adhesin
	orf19.7586	*CHT3*	*CTS1*	7.5	Chitinase
	orf19.348	*—*	*KRE6*	5.4	Beta glucan synthase
	orf19.2706	*CRH11*	*CRH1*	5.4	Putative chitin transglycosidase
	orf19.3893	*SCW11*	*SCW11*	5.3	Protein similar to glucanase
	orf19.1690	*TOS1*	*TOS1*	4.7	Alpha agglutinin anchor subunit
	orf19.2990	*XOG1*	*EXG1*	4.0	Exo-1,3-beta-glucanase
**Secreted proteins**	orf19.3839	*SAP10*	*MKC7*	11.2	Secreted aspartyl proteinase
	orf19.242	*SAP8*	*YPS1*	4.5	Secreted aspartyl proteinase

As a control, we also analyzed global expression patterns upon rapamycin treatment of the rapamycin resistant *TOR1-1/TOR1* strain [27]. Upon rapamycin exposure, the transcriptional effects observed in rapamycin treated wild type cells were largely absent and for most genes only a modest induction was observed (Table S3), indicating that the effects of rapamycin are largely mediated by inhibition of Tor1.

Overall, the transcriptional program elicited by rapamycin treatment of *C. albicans* cells reflects the role of Tor1 as a central component in a nutrient sensing signaling pathway. This role is further highlighted by the striking conservation of transcriptional programs regulated by Tor1 in *C. albicans* and *S. cerevisiae*. Moreover, it suggests a broadly conserved role for the Tor1 pathway in linking nutrient sensing to cellular growth.

### Tor1 differentially regulates morphogenesis and cellular aggregation

**Figure 1:**
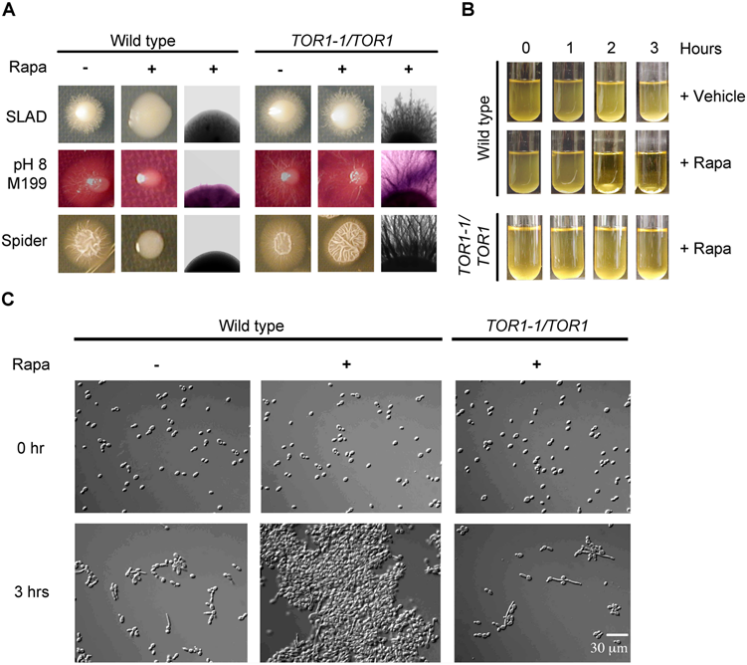
Rapamycin inhibition of Tor1 blocks filamentous growth of wild type *C. albicans* cells on agar surfaces and induces cellular aggregation and flocculation in liquid Spider medium. (A) Wild type cells and *TOR1-1/TOR1* cells were grown on SLAD, buffered alkaline M199 (pH 8.0), and Spider media plates in the absence or presence of 15 nM rapamycin and incubated for 7 days at 37°C. (B) Wild type cell culture suspensions grown in liquid Spider medium clear in the presence of 20 nM rapamycin after 2 hours of incubation at 37°C. Clearing of cell culture suspension upon rapamycin addition is not observed in *TOR1-1/TOR1* cell cultures under the same growth conditions. (C) Aliquots of wild type and *TOR1-1/TOR1* cells from (B) were examined under the microscope at the indicated times. Results shown in (A–C) are representative of at least three independent experiments.

The transcriptional regulation of hyphae-specific genes by Tor1 prompted us to investigate whether Tor1 is required for filamentous growth of *C. albicans*. Wild type cells were grown on several hyphae promoting agar media and induction of filamentation was examined in the presence or absence of sublethal concentrations of rapamycin. Rapamycin inhibited filamentation of wild type colonies grown on all media tested: SLAD, alkaline M199 (pH 8.0), and Spider media at 37°C. Rapamycin inhibited radial filament growth from the edges of colonies on all three growth media, as well as the colony wrinkling typically observed on the domes of colonies grown on Spider medium, resulting in smooth appearing colonies (Figure 1A). Rapamycin did not inhibit filamentation in the *TOR1-1/TOR1* rapamycin resistant strain, demonstrating that Tor1 inhibition blocks hyphal growth under the conditions tested (Figure 1A). In contrast, sublethal concentrations of rapamycin did not inhibit hyphal growth on YPD agar supplemented with 10% fetal bovine serum (FBS), even though wild type cells were sensitive to the fungicidal effects of higher concentrations of rapamycin under these conditions (data not shown). In conclusion, these results indicate that Tor1 is a central regulator of filamentous growth under nitrogen starvation, alkaline growth, and nutrient starvation conditions. Our results are also consistent with previous reports that showed similar rapamycin-induced inhibition of hyphal growth of *C. albicans*, *S. cerevisiae*, and *C. neoformans* [40],[41],[42].

Next, the effects of Tor1 inhibition on germ tube formation in liquid culture were examined. Wild type cells grown in either YPD liquid medium supplemented with 10% FBS or RPMI liquid medium showed robust germ tube formation after growth for one hour at 37°C. Addition of a sublethal concentration of rapamycin did not have any discernible effects on germ tube formation or elongation (data not shown). In Spider liquid culture medium, wild type cells also formed germ tubes in the presence of rapamycin. However, after two hours of incubation, cultures treated with rapamycin displayed a striking clearing of the growth culture (Figure 1B). Upon closer inspection, clearing of the culture was found to result from extensive cellular aggregation and flocculation of both yeast and hyphal cells, which was not observed with *TOR1-1/TOR1* rapamycin resistant mutant cells (Figure 1C). Remarkably, cellular aggregation was only observed with rapamycin treated wild type cells grown in Spider liquid media. Rapamycin did not elicit this phenotype in cells grown in alkaline M199 (pH 8.0), Lee's, minimal, SLAD, or YPD liquid media at 37°C, even after 7 hours of incubation (data not shown). Cells grown in modified Spider medium containing glucose or glycerol as a carbon source instead of mannitol (the carbon source typically added to Spider medium) continued to aggregate in the presence of rapamycin (data not shown), indicating that the specific carbon source utilized does not appear to contribute to rapamycin-induced cellular aggregation.

Based on these results we conclude that Tor1 has contrasting roles in regulating morphogenesis of *C. albicans* cells. On agar surfaces, Tor1 is a positive regulator of filamentation during growth in nitrogen (SLAD medium) and nutrient limited (Spider) conditions and in response to alkaline growth conditions. In contrast, Tor1 does not regulate hyphal growth in liquid cultures and under certain conditions (Spider liquid medium) Tor1 acts as a negative regulator of cellular adhesion. Interestingly, rapamycin treatment of *Candida guilliermondii* cells also results in cellular aggregation when grown in Spider medium at 37°C (Figure S1), indicating that Tor1 control of cellular adhesion might very well be conserved among other fungal species.

### Tor1 signaling regulates expression of *ALS1*, *ALS3*, *HWP1*, and *ECE1* genes encoding cell wall adhesins

To elucidate Tor1 roles in cellular aggregation, microarray analysis was performed on cells grown in Spider liquid medium (37°C) in the presence and absence of sublethal concentrations of rapamycin for 90 minutes. This analysis revealed the same pattern of downregulated genes as observed in cells grown in YPD media at 30°C (data not shown). However, the pattern of upregulated genes differed between these two conditions. During growth on Spider medium, rapamycin did not induce expression of permeases and transporters but resulted in a potent upregulation of several genes encoding GPI-anchored cell wall proteins. Among these, the adhesin genes *ALS1*, *ALS3*, and *HWP1* and the cell wall protein-coding gene *ECE1* were the most highly expressed (Table S4).

**Figure 2:**
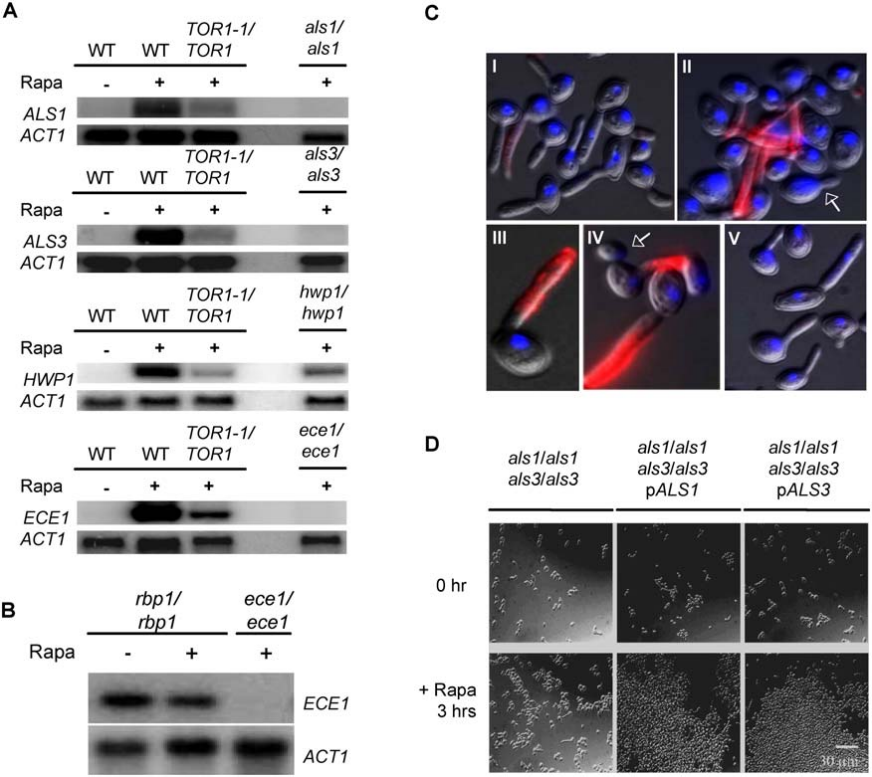
Tor1 regulates the expression of the hyphae-specific transcripts *ALS1*, *ALS3*, *HWP1*, and *ECE1* and cellular aggregation via the GPI-anchored proteins Als1 and Als3. (A) Northern analysis of *ALS1*, *ALS3*, *HWP1*, and *ECE1* in wild type and *TOR1-1/TOR1* cells grown in Spider media at 37°C and treated with 20 nM rapamycin and vehicle control for 90 minutes. (B) Expression of *ECE1* is upregulated in a *C. albicans* FKBP12 homozygous null mutant strain (*rbp1/rbp1*) grown under the same conditions as in (A). (C) Rapamycin inhibition of Tor1 results in increased Als3 at the cell wall. Als3 localization was assayed by indirect immunofluorescence using purified anti-Als3p serum in wild type and *als3/als3* strains grown in the same conditions as in (A). (I) Wild type cells+vehicle, (II) wild type cells+rapamycin, (III) magnified view of a hyphal cell from (II), (IV) magnified view of yeast and hyphal cells from (II), (V) *als3/als3* mutant cells+rapamycin. Yeast cells are indicated by arrows. Red fluor corresponds to Alexa 594 and blue to DAPI. (D) Cells of the *als1/als1 als3/als3* double mutant strain grown in Spider media at 37°C fail to aggregate upon treatment with 20 nM rapamycin. Cellular aggregation is restored when this strain is complemented by ectopic expression of either *ALS1* or *ALS3*.

Induction of *ALS1*, *ALS3*, *HWP1* and *ECE1* expression upon Tor1 inhibition was confirmed by northern analysis (Figure 2A). Surprisingly, upregulation of *ECE1* expression was also observed in a *TOR1-1/TOR1* strain, although at reduced levels compared to wild type cells treated with rapamycin. The Tor1-independent expression of *ECE1* could be due to inhibition of the *C. albicans* FKBP12 homolog Rbp1 by rapamycin. Accordingly, *ECE1* expression was modestly induced in the *rpb1/rbp1* rapamycin resistant strain lacking FKBP12 [27] irrespective of the presence of rapamycin (Figure 2B).

The cellular aggregation phenotype of rapamycin-treated cells suggests that expression of adhesin proteins in the cell wall has a phenotypic consequence. However, because rapamycin also downregulated the expression of genes involved in maintaining the translational machinery, we assayed whether induction of *ALS3* expression indeed resulted in increased Als3 protein and cell wall localization. In accord with the mRNA expression levels, rapamycin treatment increased Als3 in the cell wall of wild type cells (Figure 2C). In contrast, Als3 was undetectable in both untreated cells or in rapamycin-treated *als3/als3* mutant cells (Figure 2C). This analysis also showed that Als3 localized exclusively in hyphal cell walls, even in the presence of rapamycin (Figure 2C). We did not observe Als3 localization in yeast cell walls, suggesting that Tor1 regulation of *ALS3* expression or subsequent localization is hyphae-specific on Spider liquid medium at 37°C.

The adhesins Als1, Als3 and Hwp1 have been shown to play central roles in mediating cellular adhesion to a variety of host cell surfaces as well as mediating adhesive interactions during *C. albicans* biofilm formation (reviewed in [43]). Induction of *ALS1*, *ALS3*, *HWP1* and *ECE1* expression upon Tor1 inhibition could therefore account for the robust cell aggregation observed in cells exposed to rapamycin. Homozygous *als1/als1*, *als3/als3*, *hwp1/hwp1*, and *ece1/ece1* mutants all underwent cellular aggregation in the presence of rapamycin (data not shown). However, cells from an *als1/als1 als3/als3* homozygous double mutant strain failed to aggregate in the presence of rapamycin (Figure 2D). Complementation of this mutant strain with either the wild type *ALS1* or *ALS3* gene restored cellular aggregation (Figure 2D), providing evidence that these two adhesins function coordinately in mediating cellular aggregation upon Tor1 inhibition.

Taken together, our results show that Tor1 negatively regulates the expression of the *ALS1*, *ALS3*, *HWP1* and *ECE1* genes and, upon Tor1 inhibition, both Als1 and Als3 are functionally expressed at the cell wall and their combined actions mediate cellular aggregation of *C. albicans* cells.

### The transcriptional repressors Nrg1 and Tup1 are regulated by Tor1 signaling

**Figure 3:**
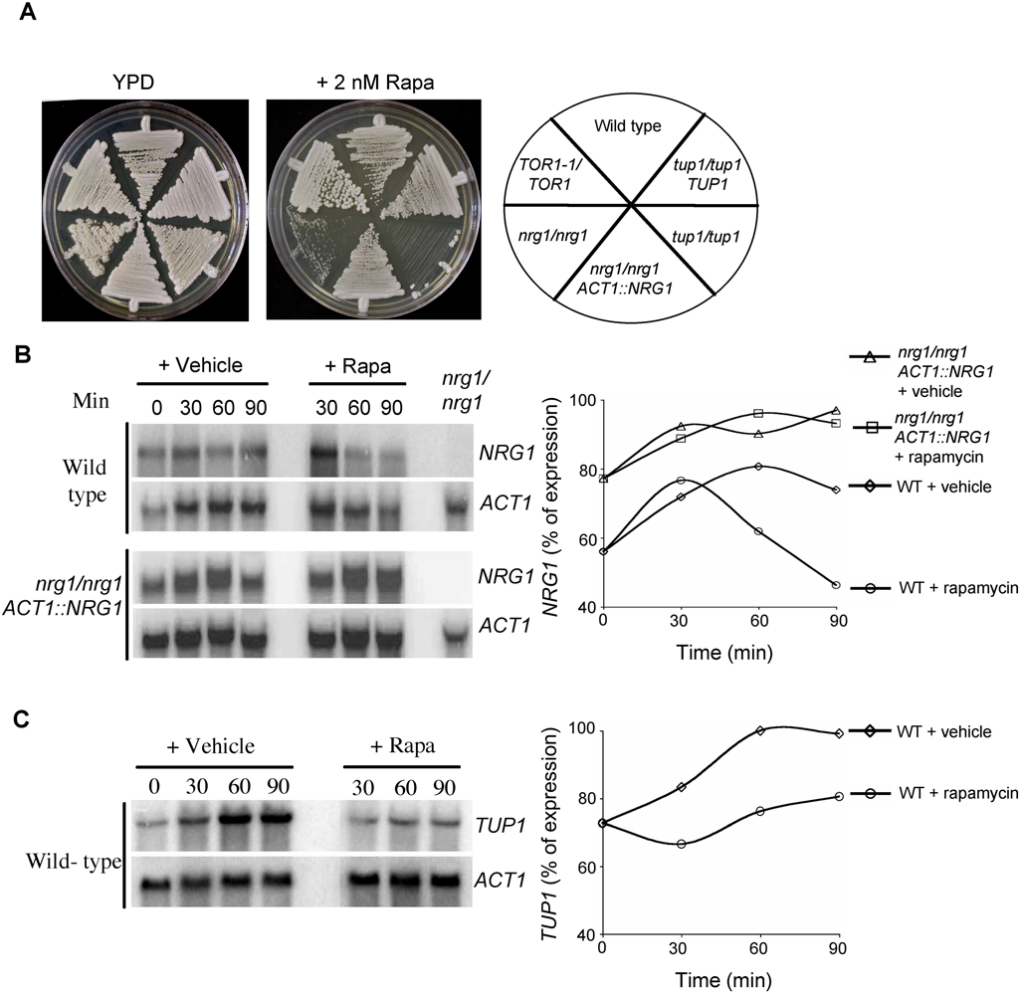
Tor1 regulates *NRG1* and *TUP1* expression. (A) Wild type (SC5314), *nrg1/nrg1*, and *tup1/tup1* mutant strains and complemented strains were grown on YPD medium with or without 2 nM rapamycin with incubation for 48 hours at 30°C and photographed. (B) *NRG1* and *ACT1* northern analysis was performed for wild type and a complemented *nrg1/nrg1* strain with a wild type *NRG1* allele expressed from the *ACT1* promoter during growth on Spider liquid medium at 37°C in the presence or absence of 20 nM rapamycin during indicated times. Northern blot signals for *NRG1* were quantified and normalized to the *ACT1* loading control. The results shown in the graph are the percentages of gene expression with the maximal level of *NRG1* expression in wild type cells treated with vehicle at 60 minutes set as 100%, and in *nrg1/nrg1 ACT1::NRG1* cells treated with rapamycin at 60 minutes set as 100%. (C) *TUP1* northern analysis of wild type cells grown under the same conditions as in (B). Hybridization signals were quantified and normalized as in (B), and plotted as percentages of gene expression with *TUP1* expression in wild type cells treated with vehicle for 60 minutes set as 100%.

*ALS1*, *ALS3*, *HWP1* and *ECE1* are hyphal specific genes whose expression is governed by a variety of signal transduction networks poised to sense environmental and nutritional cues (reviewed in [44]). In *S. cerevisiae*, loss of function mutations in Tor signaling effectors alters sensitivity of these mutants to the fungicidal effects of rapamycin. Accordingly, in order to identify transcriptional effectors downstream of Tor1 signaling in *C. albicans*, we screened homozygous null mutants in the transactivators *CPH1*, *CPH2*, *EFG1*, *TEC1*, *BCR1*, *CZF1*, *RIM101* and the repressors *NRG1*, *TUP1* and *RFG1* for those with altered rapamycin sensitivity. This screen identified the *nrg1/nrg1* and *tup1/tup1* strains as rapamycin hypersensitive (Figure 3A). Complementation of the *tup1/tup1* strain with a wild type *TUP1* allele restored a wild type level of rapamycin sensitivity (Figure 3A). Similarly ectopic expression of *NRG1* from the *ACT1* promoter in the *nrg1/nrg1* mutant strain restored wild type sensitivity to rapamycin (Figure 3A).

The rapamycin hypersensitive phenotype of the *nrg1/nrg1* and *tup1/tup1* mutant strains suggested that these transcriptional repressors could be downstream effectors of Tor1 signaling. Moreover, both repressors have been shown to negatively regulate *ALS1*, *ALS3*, *HWP1*, and *ECE1* gene expression [11],[12],[13],[14],[15],[16]. Northern analysis revealed that *NRG1* transcript levels increased in wild type cells grown in Spider medium at 37°C (Figure 3B). It is puzzling that mRNA levels of this repressor of hyphae-specific genes would increase during hyphae-inducing conditions. However, our experiments were performed by growing cells overnight in YPD liquid medium at 30°C followed by washing and resuspension of cells in Spider media. Induction of *NRG1* expression could result from shifting cells from YPD to Spider or from 30°C to 37°C. Addition of rapamycin resulted in decreased *NRG1* mRNA abundance between 30 and 90 minutes of treatment (Figure 3B). When the *NRG1* gene was expressed from the *ACT1* promoter in an *nrg1/nrg1* mutant strain, *NRG1* mRNA downregulation was no longer observed upon rapamycin treatment (Figure 3B). This finding indicates that Tor1 regulates *NRG1* transcriptionally rather than post-transcriptionally.

Unexpectedly, expression of *TUP1* also increased upon transfer of wild type cells from YPD to Spider medium at 37°C (Figure 3C). In the presence of rapamycin, induction of *TUP1* expression was curtailed (Figure 3C), indicating that Tor1 signaling is required for complete induction of this repressor.

Rapamycin downregulation of *NRG1* expression and of *TUP1* induction correlates with increased *ALS1*, *ALS3*, *HWP1* and *ECE1* mRNA abundance upon rapamycin treatment (Figures 2B, 3B, and 3C). We conclude that the *NRG1* and *TUP1* genes are both transcriptional targets of Tor1 signaling, supporting a model in which Tor1 inhibition results in decreased *NRG1* and *TUP1* mRNA levels thereby relieving *ALS1*, *ALS3*, *HWP1* and *ECE1* from transcriptional repression.

### The transcription factors Bcr1 and Efg1 are required for Tor1-mediated cell–cell aggregation and adhesin expression

**Figure 4:**
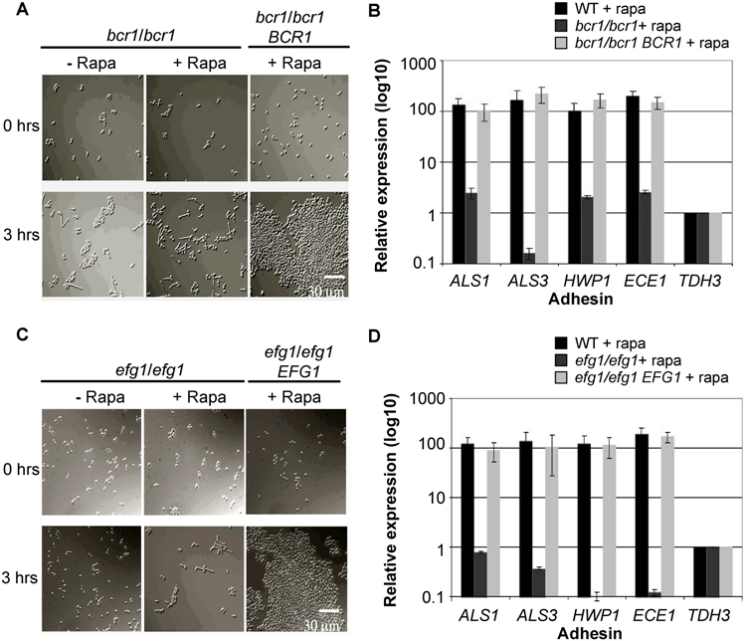
The transcription factors Bcr1 and Efg1 are required for Tor1-dependent cellular aggregation and adhesin expression. (A,C) *bcr1/bcr1*, *efg1/efg1*, and ectopically complemented mutant strains were grown in Spider medium at 37°C in the absence (−) or in the presence (+) of 20 nM rapamycin and examined microscopically at the indicated times. (B,D) *ALS1*, *ALS3*, *HWP1*, and *ECE1* mRNA expression, upon treatment with 20 nM rapamycin for 90 minutes in wild type cells grown in Spider medium at 37°C, as measured by quantitative real time PCR. Induction of *ALS1*, *ALS3*, *HWP1*, and *ECE1* expression upon rapamycin treatment is reduced in *bcr1/bcr1* and *efg1/efg1* strains and restored in complemented strains relative to untreated wild type cells under the same growth conditions. Relative expression levels represent mean ΔCt values normalized to *TDH3* expression levels and relative to expression values in untreated cells. Bars represent standard deviation for three replicates.

In *S. cerevisiae*, a common mechanism by which Tor1 regulates gene expression is by controlling the intracellular localization of transcription factors (reviewed in [33]). Accordingly, we sought to identify *C. albicans* transcription factors that might be Tor1 effectors in regulating cell–cell aggregation and adhesin expression. Our prior screen of loss of function mutants of known transcriptional regulators identified the repressors *NRG1* and *TUP1* as Tor1 downstream targets. However, we reasoned that regulation of adhesin expression and cellular aggregation is not essential for cell viability and mutations in regulators of this process might not necessarily show altered rapamycin sensitivity. This panel of mutants was re-screened for those that failed to induce cellular aggregation when grown in liquid Spider medium at 37°C in the presence of rapamycin. This screen revealed two strains, containing loss of function mutations in the regulators *BCR1* and *EFG1*, in which cells failed to aggregate upon Tor1 inhibition by rapamycin (Figure 4A and 4B). Complementation of *bcr1/bcr1* and *efg1/efg1* mutants with the wild type *BCR1* and *EFG1* genes respectively restored cellular aggregation upon rapamycin addition. We were unable to screen *nrg1/nrg1* and *tup1/tup1* mutant strains since both strains aggregated even in the absence of rapamycin (data not shown).

Expression of adhesins was also defective in both *bcr1/bcr1* and *efg1/efg1* mutant strains. We measured expression levels of *ALS1*, *ALS3*, *HWP1*, and *ECE1* by real-time quantitative PCR in wild type cells, and in *bcr1/bcr1* and *efg1/efg1* mutant strains, following rapamycin exposure for 90 minutes at 37°C. All four genes were robustly transcriptionally induced in wild type cells treated with rapamycin relative to untreated cells, in agreement with northern analysis (Figures 4C and 2A), and this induction was strongly dependent on both *BCR1* and *EFG1* (Figure 4C and 4D). We also observed residual levels of *ALS1*, *ALS3*, *HWP1*, and *ECE1* expression that were independent of Bcr1 and Efg1, suggesting some functional redundancy between Bcr1 and Efg1 or that additional Tor1 effectors are involved in this process.

Based on genetic data showing a requirement for both Bcr1 and Efg1 in rapamycin induced adhesin expression, we hypothesized that Bcr1 and Efg1 might be downstream effectors of Tor1 signaling. We measured expression levels of *BCR1* and *EFG1* by real time qPCR since mRNA levels are undetectable by northern analysis under our experimental conditions (growth in Spider media at 37°C for 30, 60 and 90 minutes).

Expression levels of *BCR1* and *EFG1* were low and remained unchanged during rapamycin treatment (data not shown), suggesting post-transcriptional regulation of Bcr1 and Efg1 activity by Tor1 (data not shown). However, we failed to detect the Bcr1 and Efg1 proteins in strains expressing Bcr1 and Efg1 with C-terminal 3XHA epitope tags. Under these conditions, it appears that the abundance of both Bcr1 and Efg1 is below the detection limit of standard techniques. Nevertheless, our genetic data implicates both Bcr1 and Efg1 as effectors of Tor1 mediated regulation of adhesin expression and cell–cell aggregation.

## Discussion

**Figure 5:**
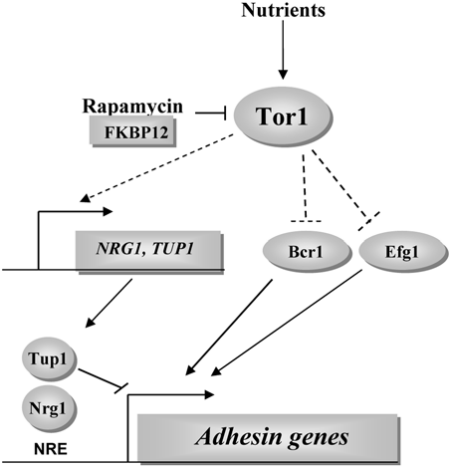
Proposed model by which Tor1 regulates *ALS1*, *ALS3*, *HWP1*, and *ECE1* expression. Scarce nutrients, perception of a signal, or rapamycin treatment results in inactivation of Tor1 leading to induction of adhesin expression and cellular aggregation. Induction of adhesin expression results from a two-fold mechanism: by direct or indirect (dashed lines) downregulation of *NRG1* and *TUP1* expression and by activation of the transactivators Bcr1 and Efg1.

The Tor protein kinase is a globally conserved constituent of a signal transduction pathway that orchestrates transcription, translation and protein degradation in eukaryotic cells in response to nutrients. The aim of this work was to assess whether Tor function has been evolutionarily conserved among the ascomycete fungal species *C. albicans* and *S. cerevisiae*. Our studies show that Tor1 mediated transcriptional regulation of translation and nitrogen starvation responses is similar between the two species, indicative of a conserved Tor1 signaling role in nutrient response pathways among ascomycetes. We have also uncovered a novel nutrient dependent role for Tor1 signaling in repressing cell–cell adhesion and expression of the adhesin genes *ALS1*, *ALS3*, *HWP1*, and *ECE1* in *C. albicans*. Tor1 mediated cell–cell adhesion is dependent on the adhesins Als1 and Als3 and adhesin expression is driven by the transcriptional activators Bcr1 and Efg1. In addition, Tor1 promotes expression of the transcriptional repressors Nrg1 and Tup1, indicating that under ample nutrient conditions, Tor1 controls adhesin expression by a dual mechanism involving repression of activators and activation of repressors as outlined in Figure 5. Cell–cell adhesion is an important virulence trait associated with *C. albicans* biofilm development and the governance of this process by Tor1 underscores the pivotal role that nutrient responsive pathways play in pathogen-associated virulence attributes.

### Tor1 and nutrient sensing in *C. albicans*

Exposure of *S. cerevisiae* cells to rapamycin triggers changes in gene expression that mimic those observed during nutrient starvation, such as transcriptional repression of genes required for ribosome biogenesis and induction of genes involved in nitrogen utilization. These and other observations have led to the model that the Tor pathway regulates growth and proliferation in response to nutrients (reviewed in [33]). Consistent with this model, our results strongly suggest that in *C. albicans*, Tor1 functions in an analogous fashion since rapamycin treatment elicited a similar nutrient scavenging transcriptional response. This response includes the coordinate downregulation of genes required for ribosome biogenesis, translation initiation and tRNA synthesis (Table S1), and concomitant induction of genes coding for amino acid transporters and peptidases necessary for nutrient acquisition and protein degradation (Table S2). Exposure to rapamycin also resulted in robust expression of nitrogen catabolite repressed (NCR) genes (Table S2) such as those encoding the high affinity ammonium permease Mep2 and the general amino acid permease Gap2. Thus, Tor1 control of the NCR response also appears to be functionally conserved. Furthermore, in *Schizosaccharomyces pombe*, the Tor1 homologue *tor2^+^* is also involved in repressing nitrogen starvation-responsive genes [45],[46] signifying that this role for Tor1 signaling is broadly conserved among fungal species.

### Tor1 regulation of filamentation

Our microarray analysis also revealed a novel role for Tor1 in regulating hyphae-specific gene transcription (Table 1). Expression of the hyphae- nduced-transcripts *ALS1*, *RBT1*, *ALS3*, *ECE1* and *SUN41* increased upon Tor1 inhibition, implicating Tor1 in governing the yeast-to-hyphae morphological transition. In accord with this line of evidence, we found that Tor1 is required for *C. albicans* filamentation on agar surfaces under a variety of hyphae-inducing conditions (Figure 1A). These results are consistent with previous reports documenting rapamycin suppression of filamentous differentiation in *S. cerevisiae*, *C. albicans*, and *C. neoformans* [40],[41],[42]. Surprisingly, rapamycin did not inhibit hyphal growth in liquid medium. This interesting and paradoxical effect suggests that Tor1 is required for contact-dependent induction of *C. albicans* hyphal growth on semi solid surfaces and perhaps during contact with host cells or extracellular matrix. In addition, we find that Tor1 is also required for hyphal growth induction during nitrogen limiting conditions (Figure 1A). Recently, there has been increasing evidence-implicating Tor1 signaling in this developmental transition. In *S. cerevisiae*, Tor1 mediated gene expression of the high affinity ammonium transporter Mep2 is controlled by the GATA transcriptional regulator Gln3 [35],[36],[38],[39]. Similarly, in *C. albicans* the Gln3 homologue regulates the expression of *MEP2* and is a likely effector of Tor1 signaling. Filamentous growth is blocked in *mep2/mep2* and *gln3/gln3* mutants under limiting nitrogen conditions, mirroring the inhibition of filamentous growth in rapamycin treated wild type cells grown on SLAD medium (Figure 1A) [47],[48],[49].

The differential regulation of filamentation by Tor1 is a reflection of the complexity inherent within the Tor1 signaling network and of its role as an integrator of diverse signals and filamentous growth.

### Regulation of cell–cell adhesion by Tor1 signaling

Another unique finding revealed during our analysis of Tor1 function in *C. albicans* is an unexpected role in regulating cell–cell adhesion. Tor1 dependent cell–cell adhesion appears to be conditional since it is only observed in liquid Spider media suggesting that it requires a signal present in this medium. This conditional regulation of cell–cell adhesion could constitute a specialized signal transduction pathway (involving Tor1) utilized by *C. albicans* to regulate cell adherence in unique niches of the mammalian host, or upon perception of nutritional or environmental signals. Nevertheless, our data strongly indicates a role for Tor1 signaling in regulating this process.

Tor1 dependent cell adherence could arise from changes in cell wall composition upon Tor1 inhibition resulting in non-specific electrostatic interactions. However, several lines of evidence support a model in which Tor1 regulates cell adherence via transcriptional regulation of cell surface adhesins. First, inhibition of Tor1 results in induced expression of genes encoding the adhesins Als1, Als3, Hwp1 and Ece1 (Table S4 and Figure 1A), all of which play complementary roles in promoting cell adhesion in *in vivo* and *in vitro* models of biofilm formation [6],[7],[8]. In particular, both Als1 and Als3, which have overlapping functions in cell adherence during biofilm development, are also required for Tor1 mediated cellular aggregation (Figure 2A and 2D) [6],[8]. Thus, Tor1 inhibits cell adhesion by promoting repression of adhesin gene expression.

Second, the finding that rapamycin induced cellular aggregation and adhesin expression is strongly dependent on the transcriptions factors Efg1 and Bcr1 (Figure 4) further strengthens our model. Both Efg1 and Bcr1 are known transactivators of *ALS1*, *ALS3*, *HWP1*, and *ECE1* gene expression and in the context of biofilm formation, Bcr1 has a well-established role in promoting cellular adhesive interactions [6],[50],[51],[52],[53],[54]. Our genetic analysis thus strongly suggests that Tor1 functions to negatively regulate Efg1 and Bcr1 activity thereby preventing induction of adhesin expression.

Our studies also implicate the adhesin transcriptional repressors Nrg1 and Tup1 in Tor1 mediated cell adhesion [11]–[16]. Rapamycin treatment of wild type cells resulted in downregulation of *NRG1* and *TUP1* mRNA expression (Figure 3B and 3C), which could result in relief of transcriptional repression at adhesin gene promoters. This model is consistent with the known hyper-flocculant phenotypes of *nrg1/nrg1* and *tup1/tup1* mutant strains. Interestingly, this level of regulation appears to be transient since Tor1 inhibition did not result in constitutive hyphal induction in contrast to that observed in *nrg1/nrg1* and *tup1/tup1* mutant strains.

Based on these studies, it is evident that Tor1 governs cellular adhesion by multiple mechanisms. One simple model consolidating our observations is that Tor1 controls *NRG1* and *TUP1* expression through Efg1 and Bcr1. Efg1 is a known transcriptional activator of *TEC1* gene expression [52], and Tec1 is necessary for *BCR1* expression [5]. However, this model is not consistent with the finding that *tec1/tec1* mutant cells continue to aggregate in the presence of rapamycin (data not shown). Furthermore, neither *bcr1/bcr1* nor *efg1/efg1* strains exhibit constitutive hyphal induction, suggesting that steady state levels of *NRG1* and *TUP1* mRNAs remain unchanged in these mutant backgrounds and arguing that Tor1 regulation of *NRG1* and *TUP1* gene expression is independent from Efg1 and Bcr1. Therefore, our results are consistent with a model that under ample nutrient conditions, Tor1 blocks cellular aggregation by promoting expression of the adhesin transcriptional repressors Nrg1 and Tup1 and by downregulating Bcr1 and Efg1 activity (Figure 5). Conversely, during nutrient limiting conditions or sensing of an environmental signal or upon rapamycin treatment, Tor1 is inactivated leading to adhesin expression and aggregation of cells, a process seminal for *C. albicans* niche colonization and biofilm formation.

Whether these events reflect a synergistic mechanism for maximal adhesin induction, or are differentially regulated by distinct signals in various host environments, requires further dissection. In summary, our results link the nutrient-responsive Tor signal transduction cascade to regulation of cell–cell adhesion in a major human fungal pathogen. Rapamycin also modulates aggregation of *C. guilliermondii* cells (Figure S1), and expression of adhesion molecules in mammalian endothelial cells [55], opening the possibility that Tor1's regulation of cell–cell adhesion might be broadly conserved among organisms including metazoans.

## Materials and Methods

### Strains and growth media

Strains used in this study are listed in Table S5. Wild type cells were grown at 30°C or 37°C on YPD (2% Bacto Peptone, 1% yeast extract and 2% dextrose), SLAD (1.7 g/L Yeast Nitrogen Base without amino acids and ammonium sulfate (Fisher scientific), supplemented with 100 µM ammonium sulfate and 2% dextrose), alkaline M199 (pH 8) (9.5 g/L Medium 199 with Earle's salts and L-glutamine and without sodium bicarbonate (Gibco), buffered with 100 mM HEPES), Spider [56], Spider without mannitol and supplemented with either 2% glucose or 2% glycerol, YPD supplemented with 10% Fetal Bovine Serum (Gibco), RPMI (RPMI 1640 liquid media with Glutamine and without sodium bicarbonate (Gibco) supplemented with 2% dextrose and buffered with 0.165 M MOPS, pH 7), Lee's [57], and SD media. Strains were grown in either solid media containing 2% agar or in liquid cultures in the presence of rapamycin (LC Laboratories) or drug vehicle (90% EtOH/10% Tween-20).

### Microarray analysis

For microarray analysis, cell cultures were grown in YPD medium at 30°C to an O.D_600_ = 0.5. Cultures were treated with either 20 nM rapamycin or drug vehicle and incubated for 60 minutes at 30°C. Total RNA was extracted using a RiboPure-Yeast RNA extraction kit (Ambion Inc) and corresponding cDNA synthesized using an AffinityScript-Multiple Temperature Reverse Transcriptase kit (Stratagene). cDNA generated from drug vehicle treated cells were used as reference and labeled with Cy3 (Amersham) and cDNA synthesized from rapamycin treated cells were labeled with Cy5 (Amersham) and used as the experimental sample. Labeled cDNA were hybridized overnight at 42°C to a 70-mer *C. albicans* AROS V1.2 oligo microarray set (OPERON technologies) printed at Duke University microarray facility. Hybridized arrays were scanned with an Axon GenePix scanner (GenePix 400B, Molecular Devices) and data extracted using GenePix Pro 4 software (Molecular Devices). Normalization and expression analysis was performed using GeneSpring (Silicon Genetics) and Excel software.

For microarray analysis of strains grown on Spider medium, cell cultures were grown in YPD liquid medium at 30°C to an O.D_600_ = 0.5. Cells were washed twice and resuspended in Spider liquid medium. Cultures were treated with either 20 nM rapamycin or drug vehicle and shaken for 90 minutes at 37°C. All microarray analyses were performed with 4 independent biological replicates.

Probability scores were calculated with GraphPad Software (http://www.graphpad.com/quickcalcs/pvalue1.cfm) using two degrees of freedom and values from a t-test on the ratio of median values, which was scaled by the standard deviation of four independent replicates. Significantly modulated genes were selected from those whose expression was above two fold higher or lower than wild type and with a p-value less than 0.05.

### Filamentation and cellular aggregation assays

Filamentation of *C. albicans* was induced by growing cells on SLAD, alkaline M199 (pH 8) and Spider agar media containing 15 nM rapamycin or drug vehicle and incubated at 37°C for 7 days. Colonies were photographed at a 4.5× magnification. For cellular aggregation assays cells were grown in YPD medium to an O.D_600_ = 0.5, washed twice and resuspended in Spider media. Cultures were treated with either 20 nM or drug vehicle, incubated at 37°C, and photographed immediately after shaking. Cells aliquots were imaged by DIC microscopy at 40× magnification using a Zeiss Axioskop 2 upright microscope.

### Northern analysis

For adhesin northern analysis, cells were grown in Spider media following the same procedure as used for cellular aggregation assays and cells treated with 20 nM rapamycin and drug vehicle for 90 minutes at 37°C. For *NRG1* and *TUP1* expression analysis, cells were similarly treated and 20 ml aliquots harvested at 0, 30, 60 and 90 minutes. Total RNA was isolated using a RiboPure-Yeast RNA extraction kit (Ambion Inc) and 30 µg of total RNA loaded onto a 1% formaldehyde agarose gel. Following transfer, membranes were hybridized to radioactive DNA probes for each specific gene (see Table S6 for primer sequences used). Hybridized probe signal was detected using a phosphoimager and quantified with Image Quant 5.2 (Molecular Dynamics) software.

### Als3 localization by indirect immunofluorescence

Localization of Als3 in hyphae of wild type and *als3/als3* strains was assayed by indirect immunofluorescence using a rabbit polyclonal antiserum raised against a recombinant Als3 N-terminus fragment that was kindly provided by Dr. Scott Filler [58]. Als3 antibodies were enriched by 3 sequential incubations of antiserum, on ice for one hour, with hyphae generated from 3×10^9^ *als3/als3* blastopores grown in RPMI at 37°C for 3 hours. Localization of Als3 was performed by growing wild type and *als3/als3* mutant strains using the same procedure as used for cell aggregation assays. Cells were treated with 20 nM rapamycin or drug vehicle for 90 minutes at 37°C. After treatment, cell cultures were fixed with 1% formaldehyde and spotted onto poly-lysine coated multi well slides. Slides were incubated with a 1∶50 dilution of enriched Als3-N antiserum and a 1∶50 dilution of anti-rabbit IgG secondary antibody conjugated to Alexa-594 (Molecular Probes). Cells were visualized with a 40× objective on a Zeiss Axioskop 2 upright microscope equipped with an LP 615 filter.

### Real time quantitative PCR (RT-qPCR)

Cells were grown in Spider media as above and treated with 20 nM rapamycin and drug vehicle for 90 minutes at 37°C. Total RNA was extracted and cDNA generated using an AffinityScript QPCR cDNA Synthesis kit (Stratagene). RT-qPCR products were obtained using 0.15 µg of cDNA, a Brilliant SYBR Green QPCR Master Mix kit (Stratagene) and a ABI-7900 light cycler. Primers used for RT-qPCR are described in [59]. Expression levels were calculated by comparative delta Ct and normalized to *TDH3*. Expression values are presented as Ct values of rapamycin treated samples relative to *TDH3* normalized Ct values of vehicle treated samples.

### Accession numbers

Information for the following *C. albicans* genes can found at the *Candida* Genome Database (CGD) Web site (http://www. candidagenome.org): *ALS1* (orf19.5741), *ALS3* (orf19.1816), *HWP1* (orf19.1321), *ECE1* (orf19.3374), *BCR1* (orf19.723), *TEC1* (orf19.5908), *NRG1* (orf19.7150), *TUP1* (orf19.6109), *EFG1* (orf19.610), *TOR1* (orf19.2290), *GAP2* (orf19.6993), *MEP2* (orf19.5672), *CAN1* (orf19.97), *CAN2* (orf19.111), *HIP1* (orf19.4940), *DIP5* (orf19.2942), *DUR3* (orf19.781), *CAR1* (orf19.3934), *GDH2* (orf19.2192), *URE2* (orf19.155), *GAT1* (orf19.1275), *GLN3* (orf19.3912), *GCN4* (orf19.1358), *RBT1* (orf19.1327), *SUN41* (orf19.3642), *SAP10* (orf19.3839), *SAP8* (orf19.242), *RFG1* (orf19.2823), *CZF1* (orf19.3127), *RBP1* (orf19.6452), *CPH1* (orf19.4433), *CPH2* (orf19.1187). Information for the *S. cerevisiae TOR1* gene (YJR066W) can found at the *Saccharomyces* Genome Database (SGD) Web site (http://www. yeastgenome.org). Microarray data sets can be found at the Gene Expression Omnibus Web site (http://www.ncbi.nlm.nih.gov/geo/) under the accession number GSE13176.

## Supporting Information

Figure S1 Rapamycin induces cellular aggregation of *Candida guilliermondii* cells. (A) Wild type (ATCC 6260) cell culture suspensions grown in liquid Spider medium flocculate in the presence of 20 nM rapamycin after 2 hours of incubation at 37°C. (B) Microscopic images of wild type cell cultures shown in (A) at 0 and 3 hours of rapamycin treatment. Results shown are representative of at least three independent experiments.
(10.96 MB TIF)

Table S1 Genes repressed by rapamycin treatment of wild type (SC5314) cells during growth in YPD at 30°C
(0.71 MB DOC)

Table S2 Upregulated genes induced by rapamycin treatment of wild type (SC5314) cells during growth in YPD at 30°C
(0.15 MB DOC)

Table S3 Differentially expressed genes induced by rapamycin treatment of a *TOR1-1/TOR1* (Rapa^R^) strain grown in YPD at 30°C
(0.10 MB DOC)

Table S4 Upregulated gene expression after rapamycin treatment of wild type cells grown in Spider liquid medium for 90 minutes at 37°C
(0.26 MB DOC)

Table S5 Strains used in this study
(0.10 MB DOC)

Table S6 Primers used for northern probe amplification
(0.06 MB DOC)
