# Supplementary figures and images for: The Protein Kinase Tor1 Regulates Adhesin Gene Expression in Candida albicans
Source: PLoS Pathog. 2009 Feb 6;5(2):e1000294. doi: 10.1371/journal.ppat.1000294 (PMC2631134; doi:10.1371/journal.ppat.1000294)

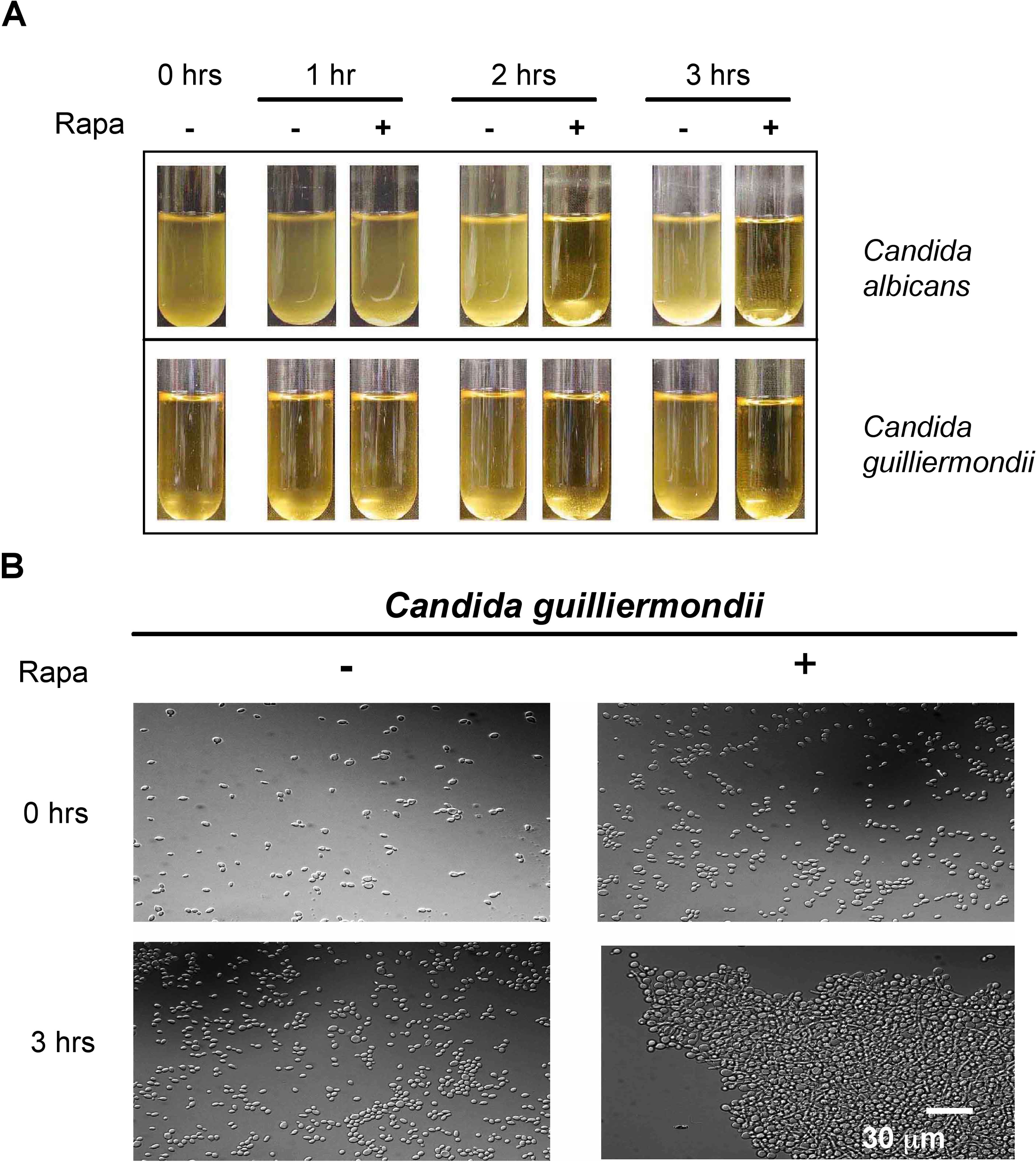

Supplement: Figure S1 — Rapamycin induces cellular aggregation of Candida guilliermondii cells. (A) Wild type (ATCC 6260) cell culture suspensions grown in liquid Spider medium flocculate in the presence of 20 nM rapamycin after 2 hours of incubation at 37°C. (B) Microscopic images of wild type cell cultures shown in (A) at 0 and 3 hours of rapamycin treatment. Results shown are representative of at least three independent experiments. (10.96 MB TIF) [file ppat.1000294.s001.tif]
