# Supplementary material for: The Protein Kinase Tor1 Regulates Adhesin Gene Expression in Candida albicans
Source: PLoS Pathog. 2009 Feb 6;5(2):e1000294. doi: 10.1371/journal.ppat.1000294 (PMC2631134; doi:10.1371/journal.ppat.1000294)
Supplement: Table S1 — Genes repressed by rapamycin treatment of wild type (SC5314) cells during growth in YPD at 30°C (0.71 MB DOC) [file ppat.1000294.s002.doc]

**Table S1**. Genes repressed by rapamycin treatment of wild-type (SC5314) cells during growth in YPD at 30°C

| **orf19_Id** | **Locus name** | ***S. cerevisiae* best hit** | **Fold change** | **p-value** | **Description** |
| --- | --- | --- | --- | --- | --- |
| **Ribosome biogenesis** | |  |  |  |  |
| orf19.2917 | *-* | *NUG1* | -12.2 | 0.0004 | Predicted component of nuclear 60S pre-ribosomes |
| orf19.7384 | *-* | *NOG1* | -6.8 | 0.0001 | Putative GTPase component of 60S ribosomal subunit |
| orf19.4815 | *YTM1* | *YTM1* | -6.1 | 0.0001 | Protein similar to *S. cerevisiae* Ytm1p |
| orf19.4029 | *-* | *SQT1* | -4.5 | 0.0002 | Predicted to aid 60S ribosomal subunit assembly |
| orf19.6975 | *YST1* | *RPS0A* | -2.5 | 0.0002 | Ribosome-associated protein |
| orf19.4336 | *RPS5* | *RPS5* | -2.4 | 0.0076 | Predicted ribosomal protein |
| orf19.6375 | *RPS20* | *RPS20* | -2.4 | 0.0015 | Predicted ribosomal protein |
| orf19.4490 | *RPL17B* | *RPL17B* | -2.3 | 0.0062 | Predicted ribosomal protein |
| orf19.6085 | *RPL16A* | *RPL16A* | -2.2 | 0.0040 | Ribosomal protein Rpl16Ap |
| orf19.827.1 | *RPL39* | *RPL36A* | -2.1 | 0.0160 | Ribosomal protein L39 |
| orf19.2425.2 | *RPL28* | *RPL28* | -2.1 | 0.0074 | Predicted ribosomal protein |
| orf19.4632 | *RPL20B* | *RPL20A* | -2.1 | 0.0009 | Predicted ribosomal protein |
| orf19.2478.1 | *-* | *RPL7A* | -2.0 | 0.0134 | Predicted ribosomal protein of the large subunit 7A |
| orf19.1700 | *RPS7A* | *RPS7A* | -2.0 | 0.0034 | Predicted ribosomal protein |
|  |  |  |  |  |  |
| **rRNA processing** | |  |  |  |  |
| orf19.4093 | *-* | *NOP7* | -100 | 0.0365 | Predicted nucleolar protein |
| orf19.3478 | *NIP7* | *NIP7* | -100 | 0.0023 | Predicted nucleolar protein |
| orf19.3990 | *-* | *RPC17* | -100 | 0.0064 | Predicted RNA polymerase III subunit C17 |
| orf19.2847 | *-* | *RPC82* | -100 | 0.0425 | Predicted RNA polymerase III subunit C82 |
| orf19.2998 | *-* | *TSR2* | -100 | 0.0030 | Predicted ORF from Assembly 19 |
| orf19.809 | *-* | *NOP12* | -100 | 0.0330 | Predicted nucleolar protein |
| orf19.7424 | *-* | *NSA2* | -100 | 0.0134 | Predicted protein of 66S pre-ribosomal particles |
| orf19.6090 | *-* | *NSR1* | -13.7 | 0.0003 | Predicted nucleolar protein |
| orf19.7050 | *NOP15* | *NOP15* | -10.2 | 0.0158 | Putative nucleolar ribosome biogenesis factor |
| orf19.3138 | *NOP1* | *NOP1* | -9.3 | 0.0000 | Nucleolar protein |
| orf19.1833 | *-* | *CBF5* | -8.9 | 0.0001 | Predicted pseudouridine synthase |
| orf19.3962 | *HAS1* | *HAS1* | -7.6 | 0.0004 | Nucleolar RNA helicase |
| orf19.501 | *-* | *NOP2* | -7.5 | 0.0006 | Predicted RNA m(5)C methyltransferase |
| orf19.4697 | *MDN1* | *MDN1* | -6.4 | 0.0000 | Predicted dynein-related AAA-type ATPase |
| orf19.7215 | *-* | *UTP10* | -5.4 | 0.0034 | Predicted component of the small subunit processome |
| orf19.526 | *NHP2* | *NHP2* | -5.3 | 0.0003 | Predicted nucleolar protein |
| orf19.7599 | *-* | *UTP5* | -4.4 | 0.0003 | Predicted component of the small subunit processome |
| orf19.7618 | *-* | *PNO1* | -4.3 | 0.0000 | Predicted nucleolar protein for pre-18S rRNA processing |
| orf19.6417 | *-* | *TSR1* | -4.2 | 0.0047 | Predicted ORF from Assembly 19 |
| orf19.5884 | *-* | *URB2* | -3.5 | 0.0002 | Predicted nucleolar rRNA processing protein |
| orf19.172 | *-* | *RPC19* | -3.4 | 0.0044 | Predicted RNA polymerase III subunit C19 |
| orf19.7255 | *RPC10* | *RPC10* | -2.7 | 0.0016 | Predicted RNA polymerase III subunit C10 |
| orf19.5436 | *-* | *UTP8* | -2.6 | 0.0012 | Predicted component of the small subunit processome |
|  |  |  |  |  |  |
| **Mitochondrial ribosome biogenesis** | | |  |  |  |
| orf19.4204 | *-* | *PET123* | -100 | 0.0114 | Predicted mitochondrial ribosomal protein of small subunit |
| orf19.7485 | *-* | *MRPL9* | -100 | 0.0287 | Mitochondrial ribosomal protein of the large subunit |
| orf19.1545 | *-* | *MRPS8* | -100 | 0.0091 | Mitochondrial ribosomal protein of the small subunit |
| orf19.5161 | *-* | *MRPL49* | -8.7 | 0.0009 | Mitochondrial ribosomal protein of the large subunit |
| orf19.5279 | *-* | *MRPL51* | -7.6 | 0.0010 | Mitochondrial ribosomal protein of the large subunit |
| orf19.397 | *-* | *MRPL28* | -6.5 | 0.0008 | Mitochondrial ribosomal protein of the large subunit |
| orf19.2214 | *-* | *MRPL7* | -5.0 | 0.0001 | Mitochondrial ribosomal protein of the large subunit |
| orf19.7012 | *-* | *MRPS16* | -5.0 | 0.0004 | Mitochondrial ribosomal protein of the small subunit |
| orf19.2275 | *-* | *MNP1* | -4.7 | 0.0000 | Putative mitochondrial ribosomal protein |
| orf19.5684 | *-* | *MRPL38* | -4.5 | 0.0003 | Mitochondrial ribosomal protein of the large subunit |
| orf19.1967 | *-* | *IMG1* | -4.5 | 0.0000 | Predicted mitochondrial ribosomal protein of large subunit |
| orf19.3797 | *-* | *MRPL11* | -4.4 | 0.0000 | Mitochondrial ribosomal protein of the large subunit |
| orf19.5698 | *-* | *MRPL1* | -4.4 | 0.0001 | Mitochondrial ribosomal protein of the large subunit |
| orf19.3559 | *-* | *MRPS35* | -4.2 | 0.0004 | Mitochondrial ribosomal protein of the small subunit |
| orf19.4018 | *-* | *RSM7* | -4.0 | 0.0006 | Predicted mitochondrial ribosomal protein |
| orf19.688 | *-* | *MRPS18* | -4.0 | 0.0006 | Mitochondrial ribosomal protein of the small subunit |
| orf19.3064 | *-* | *MRPL27* | -4.0 | 0.0006 | Mitochondrial ribosomal protein of the large subunit |
| orf19.3297 | *-* | *RSM27* | -3.9 | 0.0007 | Predicted mitochondrial ribosomal protein |
| orf19.7019 | *YML6* | *YML6* | -3.4 | 0.0004 | Mitochondrial ribosomal protein |
| orf19.6752 | *-* | *RSM10* | -2.4 | 0.0022 | Predicted mitochondrial ribosomal protein |
| orf19.3022 | *-* | *RSM24* | -2.1 | 0.0074 | Predicted mitochondrial ribosomal protein |
|  |  |  |  |  |  |
| **Translation initiation** | |  |  |  |  |
| orf19.6213 | *SUI2* | *SUI2* | -100 | 0.0427 | Putative translation initiation factor |
| orf19.4261 | *TIF5* | *TIF5* | -4.2 | 0.0004 | Putative translation initiation factor |
| orf19.5351 | *TIF11* | *TIF11* | -3.7 | 0.0014 | Predicted translation initiation factor eIF1alpha |
| orf19.407 | *GCD6* | *GCD6* | -3.7 | 0.0001 | Putative catalytic subunit of eIF2B |
| orf19.4635 | *NIP1* | *NIP1* | -3.6 | 0.0003 | Putative translation initiation factor |
| orf19.7236 | *TIF35* | *TIF35* | -3.4 | 0.0004 | Putative translation initiation factor |
| orf19.2967 | *TIF34* | *TIF34* | -3.4 | 0.0021 | Putative translation initiation factor |
| orf19.6904 | *GCN3* | *GCN3* | -3.2 | 0.0002 | Protein described as a translation initiator |
| orf19.4223 | *GCD11* | *GCD11* | -3.1 | 0.0000 | Subunit of translation initiation factor eIF2 |
| orf19.5081 | *FUN12* | *FUN12* | -3.1 | 0.0091 | Putative translation initiation factor |
| orf19.6345 | *RPG1A* | *RPG1* | -2.8 | 0.0001 | Putative translation initiation factor |
| orf19.6776 | *GCD2* | *GCD2* | -2.6 | 0.0134 | Putative translation initiation factor |
| orf19.6913 | *GCN2* | *GCN2* | -2.3 | 0.0438 | Translation initiation factor 2-alpha (eIF2alpha) kinase |
| orf19.3324 | *TIF* | *TIF1|TIF2* | -2.3 | 0.0254 | Protein similar to *S. cerevisiae* translation initiation factor |
| orf19.7613 | *HCR1* | *HCR1* | -2.0 | 0.0064 | Putative translation initiation factor |
|  |  |  |  |  |  |
| **Translation elongation** | |  |  |  |  |
| orf19.4932 | *-* | *MEF1* | -6.6 | 0.0116 | Predicted mitochondrial elongation factor |
| orf19.6047 | *TUF1* | *TUF1* | -4.9 | 0.0005 | Putative translation elongation factor |
| orf19.6208 | *MEF2* | *MEF2* | -4.0 | 0.0038 | Predicted mitochondrial elongation factor |
| orf19.3541 | *ERF1* | *SUP45* | -3.5 | 0.0172 | Putative translation release factor 1 |
|  |  |  |  |  |  |
| **tRNA synthesis** | |  |  |  |  |
| orf19.2039 | *-* | *MSF1* | -6.2 | 0.0031 | Predicted beta subunit of phenylalanyl-tRNA synthetase |
| orf19.3341 | *-* | *YDR341C* | -4.7 | 0.0000 | Putative tRNA-Arg synthetase |
| orf19.3341 | *-* | *YDR341C* | -4.7 | 0.0000 | Putative tRNA-Arg synthetase |
| orf19.500 | *-* | *GCD10* | -4.6 | 0.0330 | Predicted subunit of tRNA methyltransferase |
| orf19.518 | *-* | *NCL1* | -4.2 | 0.0009 | tRNA (cytosine-5-)-methyltransferase activity |
| orf19.4051 | *HTS1* | *HTS1* | -4.1 | 0.0009 | Putative tRNA-His synthetase |
| orf19.5685 | *THS1* | *THS1* | -3.2 | 0.0002 | Putative threonyl-tRNA synthetase |
| orf19.269 | *SES1* | *SES1* | -2.9 | 0.0023 | Seryl-tRNA synthetase |
| orf19.2560 | *CDC60* | *CDC60* | -2.9 | 0.0012 | Cytosolic leucyl tRNA synthetase |
| orf19.6749 | *KRS1* | *KRS1* | -2.9 | 0.0011 | Putative tRNA-Lys synthetase |
| orf19.5746 | *ALA1* | *ALA1* | -2.8 | 0.0042 | Putative cytoplasmic alanyl-tRNA synthetase |
| orf19.6702 | *DED81* | *DED81* | -2.7 | 0.0004 | Putative tRNA-Asn synthetase |
| orf19.5226 | *WRS1* | *WRS1* | -2.6 | 0.0010 | Putative tRNA-Trp synthetase |
| orf19.2138 | *ILS1* | *ILS1* | -2.4 | 0.0049 | Protein similar to isoleucyl-tRNA synthetase |
| orf19.7064 | *GLN4* | *GLN4* | -2.3 | 0.0026 | Putative tRNA-Gln synthetase |
| orf19.3955 | *MES1* | *MES1* | -2.2 | 0.0098 | Cytoplasmic methionyl-tRNA synthetase |
| orf19.2407 | *DPS1-1* | *DPS1* | -2.1 | 0.0032 | Putative tRNA-Asp synthetase |
|  |  |  |  |  |  |
| **Amino acid biosynthesis**  orf19.6317 | |  |  |  |  |
| orf19.5906 | *ADE6* | *ADE6* | -11.9 | 0.0022 | Enzyme of adenine biosynthesis |
| orf19.4827 | *ADE2* | *ADE2* | -9.5 | 0.0042 | Phosphoribosylaminoimadazole carboxylase |
| orf19.7484 | *ADE12* | *ADE12* | -5.0 | 0.0001 | Predicted enzyme of adenine biosynthesis |
| orf19.492 | *ADE1* | *ADE1* | -3.2 | 0.0009 | Enzyme of adenine biosynthesis |
| orf19.3554 | *ADE17* | *ADE17* | -2.1 | 0.0095 | Enzyme of adenine biosynthesis |
| orf19.3941 | *AAT1* | *-* | -9.5 | 0.0036 | Aspartate aminotransferase |
| orf19.1716 | *URA7* | *URA7* | -7.2 | 0.0000 | Predicted major CTP synthase isozyme |
| orf19.2360 | *URA3* | *URA3* | -6.3 | 0.0032 | Orotidine-5'-phosphate decarboxylase |
| orf19.1977 | *URA2* | *URA2* | -2.8 | 0.0133 | Predicted ORF from Assembly 19 |
| orf19.4177 | *-* | *URA4* | -2.5 | 0.0083 | Predicted ORF from Assembly 19 |
| orf19.4026 | *HIS5* | *HIS5* | -2.5 | 0.0079 | Predicted histidinol-phosphate aminotransferase |
| orf19.88 | *HIS1* | *HIS1* | -2.1 | 0.0108 | ATP phosphoribosyl transferase |
| orf19.4040 | *ILV5* | *ILV5* | -4.1 | 0.0024 | Protein described as ketol-acid reductoisomerase |
| orf19.5480 | *ILV3* | *ILV3* | -3.9 | 0.0012 | Putative dihydroxyacid dehydratase |
| orf19.1613 | *ILV1* | *ILV1* | -2.3 | 0.0000 | Protein described as threonine dehydratase |
| orf19.6086 | *ILV2* | *ILV2* | -2.2 | 0.0003 | Protein described as acetolactate synthase |
| orf19.3846 | *LEU4* | *LEU4* | -4.3 | 0.0116 | Putative 2-isopropylmalalate synthase |
| orf19.2618 | *LYS4* | *LYS4* | -2.1 | 0.0120 | Protein described as homoaconitase |
| orf19.2551 | *MET2* | *MET2* | -3.7 | 0.0019 | Putative homoserine acetyltransferase |
| orf19.6780 | *MET6* | *MET6* | -2.5 | 0.0153 | Putative methyltransferase |
| orf19.3911 | *MET8* | *MET8* | -2.0 | 0.0145 | Predicted bifunctional dehydrogenase |
| orf19.5838 | *SAH1* | *SAH1* | -3.2 | 0.0052 | S-adenosyl-L-homocysteine hydrolase |
| orf19.5263 | *-* | *SER2* | -6.3 | 0.0000 | Predicted ORF from Assembly 19 |
| orf19.3099 | *SER33* | *SER3* | -3.2 | 0.0057 | Predicted enzyme of amino acid biosynthesis |
| orf19.5243 | *TRP4* | *TRP4* | -3.1 | 0.0377 | Predicted enzyme of amino acid biosynthesis |
| orf19.1067 | *TRP3* | *TRP3* | -2.3 | 0.0013 | Predicted enzyme of amino acid biosynthesis |
|  |  |  |  |  |  |
| **Glycolysis and glyoxylate cycle** | | |  |  |  |
| orf19.6540 | *GPM2* |  | -10.4 | 0.0189 | Putative phosphoglycerate mutase |
| orf19.5323 | *PFK2* | *PFK2* | -6.0 | 0.0000 | Beta subunit of phosphofructokinase |
| orf19.2608 | *MDH1-3* | *-* | -3.1 | 0.0009 | Predicted malate dehydrogenase |
| orf19.3575 | *ADH5* | *-* | -2.7 | 0.0005 | Putative alcohol dehydrogenase |
| orf19.6632 | *CDC19* | *CDC19* | -2.6 | 0.0114 | Putative pyruvate kinase |
| orf19.4618 | *ACO2* | *YJL200C* | -2.5 | 0.0044 | Aconitate hydratase 2 |
| orf19.3997 | *FBA1* | *FBA1* | -2.5 | 0.0463 | Putative fructose-bisphosphate aldolase |
| orf19.3542 | *ADH1* | *ADH1* | -2.2 | 0.0228 | Alcohol dehydrogenase |
|  |  |  |  |  |  |
| **Other functions** | |  |  |  |  |
| orf19.5160 | *-* | *LEM3* | -100 | 0.0269 | Predicted protein of the plasma membrane and ER |
| orf19.3419 | *-* | *SAP190* | -100 | 0.0378 | Similar to *S. cerevisiae* Sap185p |
| orf19.2020 | *MAE1* | *MAE1* | -17.0 | 0.0000 | Malic enzyme |
| orf19.1868 | *HGT6* | *HXT6* | -16.0 | 0.0000 | Putative glucose transporter |
| orf19.4099 | *RNR22* | *-* | -12.5 | 0.0009 | Putative ribonucleoside diphosphate reductase |
| orf19.5437 | *ECM17* | *ECM17* | -11.1 | 0.0336 | Predicted enzyme of sulfur amino acid biosynthesis |
| orf19.2803 | *RHR2* | *RHR2* | -10.3 | 0.0000 | Putative glycerol 3-phosphatase |
| orf19.508 | *HEM13* | *HEM13* | -9.6 | 0.0000 | Coproporphyrinogen III oxidase |
| orf19.1575 | *QDR1* | *QDR1* | -9.3 | 0.0001 | Transporter of antibiotic resistance |
| orf19.496 | *PRS1* | *PRS3* | -8.2 | 0.0001 | Phosphoribosylpyrophosphate synthetase |
| orf19.5288 | *-* | *MSH1* | -7.7 | 0.0003 | Putative mitochondrial DNA binding protein |
| orf19.6437 | *IFE2* | *BDH1* | -7.5 | 0.0001 | Putative alcohol dehydrogenase |
| orf19.550 | *CDC23* | *CDC23* | -7.4 | 0.0420 | Putative anaphase-promoting complex component |
| orf19.5818 | *PDX3* | *PDX3* | -7.0 | 0.0000 | Predicted ORF from Assembly 19 |
| orf19.7325 | *SUR2* | *SUR2* | -7.0 | 0.0000 | Putative ceramide hydroxylase |
| orf19.3656 | *-* | *SCO1* | -6.8 | 0.0000 | Copper-binding protein |
| orf19.1030 | *-* | *COX15* | -6.2 | 0.0001 | Predicted ORF from Assembly 19 |
| orf19.406 | *-* | *FPR3* | -6.1 | 0.0002 | Predicted nucleolar peptidyl-prolyl cis-trans isomerase |
| orf19.2489 | *ERG1* | *ERG1* | -6.1 | 0.0000 | Squalene epoxidase |
| orf19.5674 | *-* | *KAP123* | -6.0 | 0.0009 | Predicted karyopherin beta 4 |
| orf19.6565 | *PGA10* | *-* | -5.7 | 0.0037 | Plasma membrane protein |
| orf19.3037 | *-* | *OXA1* | -5.6 | 0.0005 | Predicted ORF from Assembly 19 |
| orf19.7187 | *-* | *PAB1* | -5.5 | 0.0000 | Putative poly(A)-binding protein |
| orf19.4813 | *MAM33* | *MAM33* | -5.4 | 0.0008 | Predicted ORF from Assembly 19 |
| orf19.3126 | *-* | *GUA1* | -5.3 | 0.0004 | Predicted ORF from Assembly 19 |
| orf19.3325 | *-* | *CCT6* | -5.3 | 0.0044 | Predicted ORF from Assembly 19 |
| orf19.3577 | *-* | *GLG2* | -5.3 | 0.0000 | Putative glycogen synthesis initiator |
| orf19.4581 | *COQ5* | *COQ5* | -5.2 | 0.0061 | Putative methyltransferase |
| orf19.7577 | *-* | *PMH1* | -5.0 | 0.0000 | Predicted mannosyltransferase I |
| orf19.1756 | *MSS51* | *MSS51* | -4.8 | 0.0002 | Putative mRNA maturation factor |
| orf19.489 | *GPD1* | *GPD1* | -4.8 | 0.0014 | Putative glycerol-3-phosphate dehydrogenase |
| orf19.3670 | *DAP1* | *DAP1* | -4.8 | 0.0002 | Protein similar to *S. cerevisiae* Dap1p |
| orf19.2028 | *GAL1* | *GAL3* | -4.6 | 0.0202 | Galactokinase |
| orf19.5630 | *MXR1* | *MXR1* | -4.6 | 0.0018 | Methionine sulfoxide reductase |
| orf19.391 | *APA2* | *APA1* | -4.6 | 0.0000 | Protein described as ATP adenylyltransferase II |
| orf19.171 | *UPC2* | *UPC2* | -4.4 | 0.0017 | Transcription factor |
| orf19.4777 | *-* | *DBP2* | -4.3 | 0.0005 | Predicted ATP-dependent RNA helicase |
| orf19.5348 | *DAK2* | *DAK2* | -4.2 | 0.0014 | Dihydroxyacetone kinase |
| orf19.1361 | *TPS3* | *TPS3* | -4.1 | 0.0004 | Regulatory subunit of trehalose-phosphate synthase |
| orf19.5281 | *-* | *MAS6* | -3.8 | 0.0011 | Predicted ORF from Assembly 19 |
| orf19.1448 | *-* | *SCP160* | -3.8 | 0.0085 | Predicted ORF from Assembly 19 |
| orf19.5832 | *APT1* | *APT1* | -3.5 | 0.0000 | Predicted ORF from Assembly 19 |
| orf19.1626 | *HPT1* | *HPT1* | -3.4 | 0.0000 | Predicted phosphoribosyltransferase |
| orf19.388 | *-* | *DYS1* | -3.4 | 0.0001 | Predicted Deoxyhypusine synthase |
| orf19.2183 | *CAF16* | *CAF16* | -3.3 | 0.0012 | ABC family protein |
| orf19.7478 | *KRE30* | *ARB1* | -3.2 | 0.0026 | YEF3-subfamily ABC family protein |
| orf19.2524 | *-* | *COQ1* | -3.2 | 0.0003 | Predicted ORF from Assembly 19 |
| orf19.685 | *MGE1* | *MGE1* | -3.1 | 0.0009 | Predicted ORF from Assembly 19 |
| orf19.1413 | *YHM1* | *GGC1* | -3.1 | 0.0091 | Putative mitochondrial carrier protein |
| orf19.2795 | *YFH1* | *YFH1* | -3.1 | 0.0029 | Frataxin |
| orf19.198 | *-* | *LHP1* | -3.1 | 0.0007 | Putative RNA binding protein |
| orf19.7386 | *ASN1* | *ASN2|ASN1* | -3.0 | 0.0024 | Protein described as asparagine synthetase |
| orf19.5989 | *-* | *MBA1* | -3.0 | 0.0004 | Predicted ORF from Assembly 19 |
| orf19.4048 | *-* | *HRP1* | -2.9 | 0.0001 | Predicted ORF from Assembly 19 |
| orf19.18 | *DES1* | *-* | -2.9 | 0.0016 | Putative delta-4 sphingolipid desaturase |
| orf19.6074 | *IMH3* | *IMD4* | -2.9 | 0.0168 | Inosine monophosphate (IMP) dehydrogenase |
| orf19.2286 | *HBR1* | *FAP7* | -2.9 | 0.0082 | Protein involved in regulation of MTL gene expression |
| orf19.7551 | *-* | *LIA1* | -2.8 | 0.0012 | Predicted ORF from Assembly 19 |
| orf19.3675 | *ALO1* | *ALO1* | -2.8 | 0.0001 | D-Arabinono-1,4-lactone oxidase |
| orf19.6432 | *GAL7* | *GAL7* | -2.8 | 0.0012 | Predicted ORF from Assembly 19; Gcn4p-regulated |
| orf19.4381 | *-* | *AFG2* | -2.8 | 0.0012 | Predicted ORF from Assembly 19 |
| orf19.2364 | *VTC3* | *VTC2* | -2.8 | 0.0139 | Putative polyphosphate synthetase |
| orf19.2951 | *MIS11* | *MIS1* | -2.8 | 0.0117 | Predicted precursor of C1-tetrahydrofolate synthase |
| orf19.6423 | *HOM6* | *HOM6* | -2.8 | 0.0067 | Predicted homoserine dehydrogenase |
| orf19.6916 | *-* | *FBP26* | -2.8 | 0.0119 | Predicted ORF from Assembly 19 |
| orf19.2877 | *-* | *ATP11* | -2.7 | 0.0155 | Predicted ORF from Assembly 19 |
| orf19.5855 | *PDC11* | *PDC1* | -2.7 | 0.0188 | Protein similar to pyruvate decarboxylase |
| orf19.5006 | *-* | *MBP1* | -2.7 | 0.0179 | Predicted ORF from Assembly 19 |
| orf19.1201 | *-* | *GCV3* | -2.7 | 0.0067 | Predicted ORF from Assembly 19 |
| orf19.7057 | *-* | *DIA4* | -2.6 | 0.0023 | Predicted ORF from Assembly 19 |
| orf19.2711 | *-* | *GUS1* | -2.6 | 0.0007 | Predicted ORF from Assembly 19 |
| orf19.1115 | *-* | *ELP2* | -2.6 | 0.0001 | Predicted ORF from Assembly 19 |
| orf19.4537 | *-* | *GUK1* | -2.6 | 0.0003 | Predicted ORF from Assembly 19 |
| orf19.1770 | *-* | *DST1* | -2.6 | 0.0000 | Predicted ORF from Assembly 19 |
| orf19.1588 | *CYC1* | *CYC1* | -2.6 | 0.0046 | Cytochrome C |
| orf19.5942 | *-* | *FMP21* | -2.6 | 0.0182 | Predicted ORF from Assembly 19 |
| orf19.392 | *-* | *ITT1* | -2.6 | 0.0492 | Putative zinc finger protein |
| orf19.1381 | *-* | *CBP4* | -2.6 | 0.0006 | Predicted ORF from Assembly 19 |
| orf19.2956 | *-* | *LSB5* | -2.5 | 0.0032 | Predicted ORF from Assembly 19 |
| orf19.6717 | *MGM101* | *MGM101* | -2.5 | 0.0003 | Putative mitochondrial genome maintenance protein |
| orf19.719 | *-* | *FSH1* | -2.5 | 0.0001 | Predicted ORF from Assembly 19 |
| orf19.1416 | *-* | *HRD1* | -2.5 | 0.0008 | Predicted ORF from Assembly 19 |
| orf19.1404 | *COX11* | *COX11* | -2.5 | 0.0067 | Putative mitochondrial inner membrane protein |
| orf19.4022 | *-* | *DUS1* | -2.5 | 0.0139 | Predicted ORF from Assembly 19 |
| orf19.797 | *SDH4* | *-* | -2.5 | 0.0077 | Succinate dehydrogenase |
| orf19.7394 | *BAT21* | *BAT2* | -2.5 | 0.0000 | Predicted branched chain amino acid aminotransferase |
| orf19.6435 | *GDA1* | *GDA1* | -2.5 | 0.0372 | Golgi membrane GDPase |
| orf19.6082 | *-* | *FMP43* | -2.4 | 0.0045 | Predicted ORF from Assembly 19 |
| orf19.4004 | *-* | *GSF2* | -2.4 | 0.0018 | Predicted ORF from Assembly 19 |
| orf19.7052 | *-* | *CCT3* | -2.4 | 0.0257 | Predicted ORF from Assembly 19 |
| orf19.6672 | *-* | *INP52* | -2.4 | 0.0247 | Predicted Polyphosphatidylinositol phosphatase |
| orf19.4304 | *-* | *MDJ1* | -2.4 | 0.0017 | Predicted ORF from Assembly 19 |
| orf19.2774 | *GAP1* | *-* | -2.4 | 0.0003 | General amino acid permease |
| orf19.488 | *-* | *LIP5* | -2.4 | 0.0027 | Predicted ORF from Assembly 19 |
| orf19.5282 | *MEX67* | *MEX67* | -2.4 | 0.0458 | Nuclear export protein |
| orf19.336 | *-* | *-* | -2.3 | 0.0217 | Predicted ORF from Assembly 19 |
| orf19.1214 | *YAH1* | *YAH1* | -2.3 | 0.0138 | Similar to oxidoreductases |
| orf19.6126 | *-* | *MAP2* | -2.3 | 0.0153 | Predicted ORF from Assembly 19 |
| orf19.2720 | *KGD2* | *KGD2* | -2.3 | 0.0003 | Predicted dihydrolipoamide S-succinyltransferase |
| orf19.458 | *-* | *CCT4* | -2.3 | 0.0028 | Predicted ORF from Assembly 19 |
| orf19.6668 | *-* | *BCS1* | -2.3 | 0.0057 | Predicted ORF from Assembly 19 |
| orf19.6374 | *-* | *CUE1* | -2.3 | 0.0012 | Predicted ORF from Assembly 19 |
| orf19.1236 | *-* | *ATP10* | -2.3 | 0.0320 | Predicted ORF from Assembly 19 |
| orf19.3026 | *-* | *GVP36* | -2.3 | 0.0172 | Predicted ORF from Assembly 19 |
| orf19.2475 | *-* | *MAS1* | -2.3 | 0.0014 | Predicted ORF from Assembly 19 |
| orf19.410 | *PGA26* | *-* | -2.3 | 0.0011 | Putative GPI-anchored protein of unknown function |
| orf19.3363 | *-* | *ARH1* | -2.2 | 0.0069 | Predicted ORF from Assembly 19 |
| orf19.1940 | *VTC4* | *VTC4* | -2.2 | 0.0043 | Polyphosphate synthetase |
| orf19.7410 | *-* | *CAT5* | -2.2 | 0.0013 | Predicted ORF from Assembly 19 |
| orf19.3167 | *-* | *CYM1* | -2.2 | 0.0110 | Putative lysine-specific metalloprotease |
| orf19.5515 | *-* | *COX10* | -2.2 | 0.0055 | Predicted ORF from Assembly 19 |
| orf19.6640 | *-* | *CBP3* | -2.2 | 0.0028 | Predicted ORF from Assembly 19 |
| orf19.7581 | *TPS1* | *TPS1* | -2.2 | 0.0216 | Trehalose-6-phosphate synthase |
| orf19.439 | *-* | *CUS1* | -2.2 | 0.0043 | Predicted ORF from Assembly 19 |
| orf19.2422 | *-* | *MHR1* | -2.2 | 0.0069 | Predicted ORF from Assembly 19 |
| orf19.4195.1 | *ARC1* | *ARC1* | -2.2 | 0.0157 | Predicted ORF from Assembly 19 |
| orf19.2363 | *FCA1* | *FCY1* | -2.2 | 0.0007 | Cytosine deaminase |
| orf19.7445 | *-* | *FMP31* | -2.2 | 0.0130 | Predicted ORF from Assembly 19 |
| orf19.3268 | *-* | *VID24* | -2.1 | 0.0010 | Predicted ORF from Assembly 19 |
| orf19.1609 | *-* | *YKL056C* | -2.1 | 0.0093 | Putative protein that associates with ribosomes |
| orf19.411 | *-* | *KRI1* | -2.1 | 0.0344 | Predicted ORF from Assembly 19 |
| orf19.6155 | *-* | *-* | -2.1 | 0.0014 | Similar to GTPase regulators |
| orf19.5493 | *-* | *CDC9* | -2.1 | 0.0009 | Predicted ORF from Assembly 19 |
| orf19.3038 | *GSP1* | *GSP1* | -2.1 | 0.0168 | Small RAN G-protein |
| orf19.4560 | *TPS2* | *TPS2* | -2.1 | 0.0043 | Trehalose-6-phosphate (Tre6P) phosphatase |
| orf19.6987 | *-* | *BFR1* | -2.1 | 0.0131 | Predicted ORF from Assembly 19 |
| orf19.6257 | *-* | *DNM1* | -2.0 | 0.0125 | Predicted ORF from Assembly 19 |
| orf19.6822 | *GLT1* | *GLT1* | -2.0 | 0.0009 | Putative NAD(+)-dependent glutamate synthase |
| orf19.6880 | *-* | *DCN1* | -2.0 | 0.0000 | Predicted ORF from Assembly 19 |
| orf19.898 | *-* | *COG4* | -2.0 | 0.0115 | Predicted ORF from Assembly 19 |
| orf19.1291 | *HEM2* | *HEM2* | -2.0 | 0.0216 | Predicted porphobilinogen synthase |
| orf19.793 | *ABZ1* | *ABZ1* | -2.0 | 0.0004 | Predicted para-aminobenzoate (PABA) synthase |
| orf19.3438 | *CAK1* | *-* | -2.0 | 0.0107 | Monomeric CDK-activating kinase (CAK) |
|  |  |  |  |  |  |
| **Unknown function** | |  |  |  |  |
| orf19.2798 | *-* | *SCJ1* | -100 | 0.0492 | Predicted ORF from Assembly 19 |
| orf19.6623 | *-* | *YDR332W* | -100 | 0.0415 | Putative helicase |
| orf19.6973 | *-* | *ELF1* | -100 | 0.0097 | Predicted ORF from Assembly 19 |
| orf19.4479 | *-* | *-* | -100 | 0.0299 | Predicted ORF from Assembly 19 |
| orf19.322 | *-* | *YJL010C* | -100 | 0.0029 | Predicted ORF from Assembly 19 |
| orf19.6911 | *-* | *-* | -100 | 0.0232 | Predicted ORF from Assembly 19 |
| orf19.2259 | *-* | *-* | -100 | 0.0054 | Predicted ORF from Assembly 19 |
| orf19.4121 | *-* | *-* | -100 | 0.0081 | Predicted ORF from Assembly 19 |
| orf19.7345 | *-* | *-* | -100 | 0.0440 | Predicted ORF from Assembly 19 |
| orf19.6391 | *-* | *YGR205W* | -100 | 0.0225 | Predicted ORF from Assembly 19 |
| orf19.7320 | *-* | *-* | -100 | 0.0111 | Predicted ORF from Assembly 19 |
| orf19.2899 | *LIP7* | *-* | -100 | 0.0037 | Predicted lipase |
| orf19.3573 | *-* | *-* | -100 | 0.0394 | Predicted ORF from Assembly 19 |
| orf19.6156 | *-* | *PEX6* | -100 | 0.0491 | Predicted ORF from Assembly 19 |
| orf19.4105 | *-* | *YER093C-A* | -100 | 0.0315 | Predicted ORF from Assembly 19 |
| orf19.3545 | *-* | *-* | -100 | 0.0079 | Predicted ORF from Assembly 19 |
| orf19.5973 | *-* | *-* | -35.3 | 0.0186 | Predicted ORF from Assembly 19 |
| orf19.5812 | *-* | *PHB2* | -14.0 | 0.0325 | Predicted ORF from Assembly 19 |
| orf19.3810 | *-* | *YOR051C* | -14.0 | 0.0015 | Predicted ORF from Assembly 19 |
| orf19.6676 | *-* | *MTD1* | -13.2 | 0.0003 | Predicted ORF from Assembly 19 |
| orf19.7011 | *-* | *DPH5* | -12.1 | 0.0010 | Predicted ORF from Assembly 19 |
| orf19.7664 | *-* | *RRP12* | -11.2 | 0.0047 | Predicted ORF from Assembly 19 |
| orf19.4587 | *-* | *YCR087C-A* | -10.4 | 0.0001 | Predicted ORF from Assembly 19 |
| orf19.512 | *-* | *HGH1* | -8.1 | 0.0000 | Predicted ORF from Assembly 19 |
| orf19.7459 | *-* | *KRE33* | -7.5 | 0.0193 | Predicted ORF from Assembly 19 |
| orf19.6816 | *-* | *YBR238C* | -6.9 | 0.0000 | Predicted ORF from Assembly 19 |
| orf19.4580 | *-* | *YJR096W* | -6.8 | 0.0002 | Predicted ORF from Assembly 19 |
| orf19.5967 | *-* | *-* | -6.7 | 0.0001 | Predicted ORF from Assembly 19 |
| orf19.3649 | *FGR44* | *RBS1* | -6.5 | 0.0238 | Predicted ORF from Assembly 19 |
| orf19.5356 | *-* | *FES1* | -6.4 | 0.0006 | Predicted ORF from Assembly 19 |
| orf19.4846 | *-* | *YNL313C* | -6.3 | 0.0253 | Predicted ORF from Assembly 19 |
| orf19.6418 | *-* | *YGR210C* | -6.0 | 0.0007 | Predicted ORF from Assembly 19 |
| orf19.7107 | *-* | *YDL063C* | -5.9 | 0.0009 | Predicted ORF from Assembly 19 |
| orf19.2068 | *-* | *YJL122W* | -5.6 | 0.0002 | Predicted ORF from Assembly 19 |
| orf19.4617 | *-* | *-* | -5.5 | 0.0418 | Predicted ORF from Assembly 19 |
| orf19.633 | *-* | *MAK3* | -5.5 | 0.0001 | Predicted ORF from Assembly 19 |
| orf19.5136 | *-* | *-* | -5.2 | 0.0211 | Putative methyltransferase |
| orf19.904 | *-* | *YGR017W* | -5.1 | 0.0002 | Predicted ORF from Assembly 19 |
| orf19.1956 | *-* | *-* | -5.1 | 0.0145 | Predicted ORF from Assembly 19 |
| orf19.4836 | *-* | *-* | -5.0 | 0.0154 | Predicted ORF from Assembly 19 |
| orf19.5627 | *-* | *-* | -4.7 | 0.0004 | Predicted ORF from Assembly 19 |
| orf19.2324 | *-* | *-* | -4.6 | 0.0298 | Predicted ORF from Assembly 19 |
| orf19.6600 | *-* | *UBA4* | -4.5 | 0.0000 | Predicted ORF from Assembly 19 |
| orf19.1546 | *-* | *YLR193C* | -4.4 | 0.0210 | Predicted ORF from Assembly 19 |
| orf19.2261 | *-* | *-* | -4.4 | 0.0024 | Predicted ORF from Assembly 19 |
| orf19.951 | *-* | *HSH49* | -4.4 | 0.0230 | Predicted ORF from Assembly 19 |
| orf19.679 | *-* | *-* | -4.3 | 0.0008 | Predicted ORF from Assembly 19 |
| orf19.4929 | *FGR39* | *-* | -4.3 | 0.0235 | Predicted ORF from Assembly 19 |
| orf19.6736 | *-* | *-* | -4.2 | 0.0267 | Predicted ORF from Assembly 19 |
| orf19.665 | *-* | *FMP38* | -4.2 | 0.0241 | Predicted ORF from Assembly 19 |
| orf19.3291 | *NEP1* | *EMG1* | -4.1 | 0.0010 | Predicted ORF from Assembly 19 |
| orf19.6710 | *-* | *HMT1* | -4.1 | 0.0003 | Predicted ORF from Assembly 19 |
| orf19.6853 | *-* | *-* | -4.0 | 0.0116 | Predicted ORF from Assembly 19 |
| orf19.3422 | *-* | *-* | -4.0 | 0.0007 | Predicted ORF from Assembly 19 |
| orf19.3312 | *FMP27* | *YLR454W* | -4.0 | 0.0008 | Predicted ORF from Assembly 19 |
| orf19.240 | *-* | *FMP29* | -3.8 | 0.0000 | Predicted ORF from Assembly 19 |
| orf19.4447 | *-* | *FMP18* | -3.4 | 0.0004 | Predicted ORF from Assembly 19 |
| orf19.5433 | *YMC1* | *YMC1* | -3.2 | 0.0072 | Predicted ORF from Assembly 19 |
| orf19.3357 | *-* | *FMP36* | -3.2 | 0.0055 | Predicted ORF from Assembly 19 |
| orf19.2953 | *-* | *FYV4* | -3.1 | 0.0143 | Predicted ORF from Assembly 19 |
| orf19.2948 | *-* | *TOM20* | -2.9 | 0.0002 | Predicted ORF from Assembly 19 |
| orf19.1462 | *-* | *SNO1* | -2.8 | 0.0233 | Predicted ORF from Assembly 19 |
| orf19.4624 | *-* | *SMP2* | -2.7 | 0.0022 | Predicted ORF from Assembly 19 |
| orf19.1252 | *HRT2* | *YMR027W* | -2.6 | 0.0102 | Predicted ORF from Assembly 19 |
| orf19.1788 | *YME1* | *YME1* | -2.5 | 0.0082 | Predicted ORF from Assembly 19 |
| orf19.3696 | *-* | *XKS1* | -2.3 | 0.0217 | Predicted ORF from Assembly 19 |
