# Supplementary material for: The Protein Kinase Tor1 Regulates Adhesin Gene Expression in Candida albicans
Source: PLoS Pathog. 2009 Feb 6;5(2):e1000294. doi: 10.1371/journal.ppat.1000294 (PMC2631134; doi:10.1371/journal.ppat.1000294)
Supplement: Table S2 — Upregulated genes induced by rapamycin treatment of wild type (SC5314) cells during growth in YPD at 30°C (0.15 MB DOC) [file ppat.1000294.s003.doc]

**Table S2**. Upregulated features induced by rapamycin treatment of wild-type (SC5314) cells during growth in YPD at 30°C

| **orf19 Id** | **Locus name** | ***S. cerevisiae* best hit** | **Fold change** | **p-value** | **Description** |
| --- | --- | --- | --- | --- | --- |
|  |  |  |  |  |  |
| **Permeases and transporters** | | |  |  |  |
| orf19.6249 | *HAK1* | *-* | 60.8 | 0.0175 | Similar to potassium transporters |
| orf19.6993 | *GAP2* | *GAP1* | 60.0 | 0.0069 | Amino acid permease |
| orf19.2602 | *OPT1* | *OPT1* | 54.6 | 0.0397 | Oligopeptide transporter |
| orf19.5672 | *MEP2* | *MEP2* | 52.0 | 0.0011 | Ammonium permease |
| orf19.111 | *CAN2* | *CAN1* | 47.9 | 0.0368 | Arginine permease |
| orf19.4531 | *-* | *YOL075C* | 34.2 | 0.0408 | PDR-subfamily ABC transporter |
| orf19.97 | *CAN1* | *-* | 32.6 | 0.0037 | Arginine permease |
| orf19.2946 | *HNM4* | *-* | 18.2 | 0.0175 | Putative choline permease |
| orf19.2003 | *HNM1* | *-* | 13.4 | 0.0006 | Predicted choline transporter |
| orf19.7566 | *-* | *GNP1* | 12.2 | 0.0471 | Predicted glutamine permease |
| orf19.6656 | *DUR3* | *-* | 9.1 | 0.0198 | Polyamine transporter |
| orf19.4940 | *HIP1* | *HIP1* | 7.0 | 0.0001 | Predicted histidine permease |
| orf19.7100 | *-* | *AVT1* | 5.9 | 0.0317 | Predicted amino acid vacuolar transporter |
| orf19.5170 | *ENA21* | *-* | 5.0 | 0.0043 | Similar to *S. cerevisiae* sodium transporter |
| orf19.2942 | *DIP5* | *DIP5* | 4.9 | 0.0099 | Putative dicarboxylic amino acid permease |
| orf19.6948 | *CCC1* | *CCC1* | 4.8 | 0.0227 | Predicted manganese transporter |
| orf19.2072 | *-* | *HNM1* | 4.7 | 0.0431 | Predicted choline transporter |
| orf19.5759 | *SNQ2* | *SNQ2* | 4.5 | 0.0004 | Putative *S. cerevisiae* Snq2p transporter |
| orf19.5100 | *MLT1* | *-* | 4.3 | 0.0057 | Vacuolar membrane transporter |
| orf19.1357 | *FCY21* | *FCY2* | 4.0 | 0.0103 | Putative purine-cytosine permease |
|  |  |  |  |  |  |
| **Nitrogen starvation response** | | |  |  |  |
| orf19.7029 | *-* | *GUD1* | 27.5 | 0.0231 | Predicted guanine deaminase |
| orf19.2065 | *-* | *DAL2* | 23.6 | 0.0208 | Predicted allantoicase |
| orf19.3121 | *-* | *URE2* | 21.2 | 0.0055 | Predicted nitrogen catabolite repressor |
| orf19.3418 | *-* | *CAR1* | 17.7 | 0.0367 | Predicted arginase |
| orf19.2686 | *-* | *CPS1* | 6.0 | 0.0005 | Putative carboxypeptidase |
| orf19.2192 | *GDH2* | *GDH2* | 4.0 | 0.0058 | Putative NAD-specific glutamate dehydrogenase |
| orf19.1275 | *GAT1* | *GAT1* | 3.1 | 0.049 | Transcriptional regulator of nitrogen utilization |
|  |  |  |  |  |  |
| **Drug resistance** | |  |  |  |  |
| orf19.3120 | *-* | *-* | 12.2 | 0.0094 | Putative PDR-subfamily ABC transporter |
| orf19.7306 | *-* | *YPR127W* | 8.7 | 0.0361 | Protein of aldo-keto reductase family |
| orf19.1783 | *-* | *YOR1* | 7.3 | 0.0025 | Predicted ABC family transporter |
| orf19.304 | *-* | *-* | 6.4 | 0.0495 | Putative MDR transporter |
|  |  |  |  |  |  |
| **Amino acid and protein degradation** | | |  |  |  |
| orf19.1847 | *ARO10* | *ARO10* | 37.3 | 0.0100 | Pyruvate decarboxylase |
| orf19.4135 | *PRC2* | *-* | 16.7 | 0.0430 | Carboxypeptidase |
| orf19.539 | *LAP3* | *LAP3* | 12.5 | 0.0281 | Aminopeptidase |
| orf19.5197 | *APE2* | *APE2* | 7.2 | 0.0226 | Leucine aminopeptidase |
| orf19.1628 | *LAP41* | *LAP4* | 6.5 | 0.0229 | Predicted vacuolar aminopeptidase |
| orf19.3591 | *APE3* | *APE3* | 4.5 | 0.0105 | Predicted vacuolar aminopeptidase Y |
|  |  |  |  |  |  |
| **Transcription regulation** | |  |  |  |  |
| orf19.5338 | *GAL4* | *GAL4* | 5.5 | 0.0309 | Transcription factor |
| orf19.1358 | *GCN4* | *GCN4* | 4.3 | 0.0142 | Transactivator of amino acid starvation response |
