# Supplementary material for: The Protein Kinase Tor1 Regulates Adhesin Gene Expression in Candida albicans
Source: PLoS Pathog. 2009 Feb 6;5(2):e1000294. doi: 10.1371/journal.ppat.1000294 (PMC2631134; doi:10.1371/journal.ppat.1000294)
Supplement: Table S3 — Differentially expressed genes induced by rapamycin treatment of a TOR1-1/TOR1 (RapaR) strain grown in YPD at 30°C (0.10 MB DOC) [file ppat.1000294.s004.doc]

**Table S3.** Differentially expressed features induced by rapamycin treatment of a *TOR1-1/TOR1* (Rapa^R^) strain grown in YPD at 30°C

**Downregulated features**

| **orf19_Id** | **Locus name** | ***S. cerevisiae* best hit** | **Fold change** | **p-value** | **Description** |
| --- | --- | --- | --- | --- | --- |
| orf19.396 | *-* | *EAF6* | -35.0 | 0.0430 | Predicted ORF from Assembly 19 |
| orf19.3670 | *GAL1* | *GAL3* | -3.2 | 0.0212 | Galactokinase |
| orf19.2947 | *SNZ1* | *SNZ1* | -2.8 | 0.0219 | Similar to stationary-phase-related proteins |
| orf19.633 | *-* | *-* | -2.8 | 0.0220 | Putative methyltransferase |
| orf19.3672 | *GAL10* | *GAL10* | -2.1 | 0.0000 | Putative UDP-glucose 4-epimerase |
| orf19.3554 | *AAT1* | *-* | -2.1 | 0.0333 | Unknown function |

**Upregulated features**

| **orf19_Id** | **Locus name** | ***S. cerevisiae* best hit** | **Fold change** | **p-value** | **Description** |
| --- | --- | --- | --- | --- | --- |
| orf19.2602 | *OPT1* | *OPT1* | 9.0 | 0.0041 | Oligopeptide transporter |
| orf19.341 | *-* | *-* | 8.8 | 0.0013 | Predicted ORF from Assembly 19 |
| orf19.780 | *-* | *DUR1,2* | 8.6 | 0.0029 | Predicted ORF from Assembly 19 |
| orf19.6053 | *CIS2* | *ECM38* | 5.1 | 0.0013 | Putative regulator of cell wall biogenesis |
| orf19.3926 | *-* | *RNY1* | 4.3 | 0.0030 | Predicted ORF from Assembly 19 |
| orf19.5641 | *CAR2* | *CAR2* | 3.6 | 0.0021 | Alkaline upregulated |
| orf19.1327 | *RBT1* | *-* | 3.3 | 0.0091 | Putative cell wall protein |
| orf19.7106 | *-* | *VPS70* | 3.3 | 0.0043 | Predicted ORF from Assembly 19 |
| orf19.993 | *-* | *-* | 3.2 | 0.0064 | Predicted ORF from Assembly 19 |
| orf19.2114 | *-* | *-* | 3.1 | 0.0181 | Predicted ORF from Assembly 19 |
| orf19.1847 | *ARO10* | *ARO10* | 3.1 | 0.0003 | Protein described as pyruvate decarboxylase |
| orf19.5741 | *ALS1* | *-* | 3.0 | 0.0034 | Adhesin |
| orf19.1473 | *-* | *-* | 2.8 | 0.0486 | Predicted ORF from Assembly 19 |
| orf19.3934 | *CAR1* | *CAR1* | 2.6 | 0.0034 | Putative arginase |
| orf19.5197 | *APE2* | *APE2* | 2.6 | 0.0003 | Protein repressed during the mating process |
| orf19.4716 | *GDH3* | *GDH3* | 2.5 | 0.0165 | Putative NADP-glutamate dehydrogenase |
| orf19.4445 | *-* | *-* | 2.5 | 0.0151 | Predicted ORF from Assembly 19 |
| orf19.4082 | *DDR48* | *-* | 2.4 | 0.0009 | Stress-associated protein |
| orf19.6570 | *NUP* | *-* | 2.4 | 0.0281 | Nucleoside permease |
| orf19.5773 | *-* | *YOL057W* | 2.4 | 0.0067 | Predicted ORF from Assembly 19 |
| orf19.5250 | *-* | *-* | 2.3 | 0.0020 | Predicted ORF from Assembly 19 |
| orf19.789 | *PYC2* | *PYC2* | 2.2 | 0.0178 | Putative pyruvate carboxylase |
| orf19.6656 | *DUR3* | *-* | 2.2 | 0.0001 | Putative urea transporter |
| orf19.3974 | *PUT2* | *PUT2* | 2.2 | 0.0244 | Alkaline upregulated |
| orf19.7098 | *-* | *YKL070W* | 2.2 | 0.0071 | Predicted ORF from Assembly 19 |
| orf19.889 | *THI20* | *THI20* | 2.2 | 0.0092 | Putative phosphomethylpyrimidine kinase |
| orf19.4933 | *-* | *-* | 2.1 | 0.0298 | Predicted ORF from Assembly 19 |
| orf19.7522 | *-* | *-* | 2.1 | 0.0000 | Predicted ORF from Assembly 19 |
| orf19.2397 | *-* | *-* | 2.1 | 0.0281 | Predicted ORF from Assembly 19 |
| orf19.5992 | *-* | *-* | 2.1 | 0.0029 | Predicted zinc-finger protein |
| orf19.6757 | *-* | *GCY1* | 2.1 | 0.0030 | Predicted ORF from Assembly 19 |
| orf19.434 | *PRD1* | *PRD1* | 2.0 | 0.0004 | Putative Zinc metalloendopeptidase |
| orf19.6402 | *CYS3* | *CYS3* | 2.0 | 0.0117 | Putative enzyme of amino acid biosynthesis |
