# Supplementary material for: The Protein Kinase Tor1 Regulates Adhesin Gene Expression in Candida albicans
Source: PLoS Pathog. 2009 Feb 6;5(2):e1000294. doi: 10.1371/journal.ppat.1000294 (PMC2631134; doi:10.1371/journal.ppat.1000294)
Supplement: Table S4 — Upregulated gene expression after rapamycin treatment of wild type cells grown in Spider liquid medium for 90 minutes at 37°C (0.26 MB DOC) [file ppat.1000294.s005.doc]

**Table S4.** Upregulated gene expression after rapamycin treatment of wild-type cells grown in Spider liquid medium for 90 minutes at 37°C.

| **orf19_Id** | **Locus name** | ***S. cerevisiae* best hit** | **Fold change** | **p-value** | **Description** |
| --- | --- | --- | --- | --- | --- |
| **Adhesion** |  |  |  |  |  |
| orf19.3374 | *ECE1* | *-* | 519.2 | 0.0176 | GPI anchored protein |
| orf19.1816 | *ALS3* | *FLO1* | 180.1 | 0.0033 | Adhesin |
| orf19.5741 | *ALS1* | *FLO9* | 20.7 | 0.0059 | Adhesin |
| orf19.548 | *CDC10* | *CDC10* | 5.5 | 0.0443 | Septin |
| orf19.4565 | *BGL2* | *BGL2* | 3.8 | 0.0098 | Beta-1,3 glucan transferase |
| orf19.5908 | *TEC1* | *TEC1* | 3.8 | 0.0042 | TEA/ATTS transcription factor |
| orf19.1490 | *MSB2* | *MSB2* | 3.4 | 0.0125 | Cell surface flocculin |
|  |  |  |  |  |  |
| **Cell wall organization** | | |  |  |  |
| orf19.6081 | *PHR2* | *GAS1* | 10.5 | 0.0414 | 1,3-beta-glucanosyltransferase |
| orf19.5760 | *IHD1* | *SRP40* | 9.7 | 0.0082 | Putative GPI-anchored protein |
| orf19.5073 | *DPM1* | *DPM1* | 7.6 | 0.0001 | Dolichol phosphate mannose synthase |
| orf19.1690 | *TOS1* | *TOS1* | 7.1 | 0.0210 | Putative alpha agglutinin anchor subunit |
| orf19.3642 | *SUN41* | *SIM1* | 6.0 | 0.0019 | Putative cell wall protein |
| orf19.5244 | *MCD4* | *MCD4* | 5.4 | 0.0289 | Required for (GPI) anchor synthesis |
| orf19.3732 | *ERG25* | *ERG25* | 5.1 | 0.0323 | Putative C-4 methyl sterol oxidase |
| orf19.4265 | *UAP1* | *QRI1* | 4.4 | 0.0541 | Acetylglucosamine pyrophosphorylase |
| orf19.1738 | *UGP1* | *UGP1* | 3.3 | 0.0198 | UTP-glucose-1-phosphaturidyl transferase |
| orf19.2706 | *CRH11* | *RH1* | 3.2 | 0.0029 | Predicted glycosyl hydrolase |
| orf19.4109 | *PMT4* | *PMT4* | 3.0 | 0.0048 | Protein mannosyltransferase |
|  |  |  |  |  |  |
| **Vessicle trafficking** | | |  |  |  |
| orf19.4579 | *ERV29* | *ERV29* | 6.1 | 0.0194 | Similar to *S. cerevisiae* Erv29p |
| orf19.6176 | *SEC61* | *SEC61* | 5.8 | 0.0039 | Similar to *S. cerevisiae* Sec61p |
| orf19.2937 | *PMM1* | *SEC53* | 4.8 | 0.0781 | Similar to *S. cerevisiae* Sec53p |
| orf19.4180 | *SEC72* | *SEC72* | 4.3 | 0.0407 | ER protein-translocation complex member |
| orf19.491 | *SEC63* | *SEC63* | 4.3 | 0.0040 | Similar to *S. cerevisiae* Sec63p |
| orf19.3052 | *YPT1* | *YPT1* | 4.2 | 0.0494 | Functional homolog of S. cerevisiae Ypt1p |
| orf19.5618 | *VPS45* | *VPS45* | 4.1 | 0.0293 | Predicted vacuolar sorting protein |
| orf19.4732 | *SEC24* | *SEC24* | 3.8 | 0.0051 | Similar to *S. cerevisiae* Sec24p |
| orf19.3409 | *SEC12* | *SEC12* | 3.0 | 0.0064 | Similar to *S. cerevisiae* Sec12p |
|  |  |  |  |  |  |
| **Copper and iron utilization** | | |  |  |  |
| orf19.3646 | *CTR1* | *CTR1* | 12.8 | 0.0368 | Copper transporter |
| orf19.1715 | *IRO1* | *YJL225C* | 7.2 | 0.0342 | Putative transcription factor |
| orf19.3940.1 | *CUP1* | *-* | 5.5 | 0.0433 | Copper ion binding protein. |
| orf19.7068 | *MAC1* | *MAC1* | 4.5 | 0.0277 | Transcriptional regulator of CTR1 |
| orf19.543 | *FUM1* | *FUM1* | 3.4 | 0.0111 | Putative fumarate hydratase |
|  |  |  |  |  |  |
| **Drug resistance** | |  |  |  |  |
| orf19.4805 | *RSN1* | *RSN1* | 7.5 | 0.0060 | Drug pump (Tunicamycin) |
| orf19.4082 | *DDR48* | *DDR48* | 6.3 | 0.0099 | Stress-associated protein |
| orf19.7306 | *-* | *YPR127W* | 5.7 | 0.0383 | Protein of aldo-keto reductase family |
| orf19.5170 | *ENA21* | *ENA2* | 5.1 | 0.0057 | Putative sodium transporter |
| orf19.2170 | *-* | *PHM7* | 5.0 | 0.0108 | Putative drug transporter |
| orf19.600 | *TRK1* | *TRK2* | 4.1 | 0.0514 | Putative low-affinity potassium transporter |
| orf19.4531 | *-* | *YOL075C* | 3.5 | 0.0310 | Putative PDR-subfamily ABC transporter |
| orf19.5759 | *SNQ2* | *SNQ2* | 3.1 | 0.0297 | Similar to *S. cerevisiae* Snq2p transporter |
| orf19.6812 | *PMT2* | *PMT2* | 2.7 | 0.0223 | Protein mannosyltransferase |
|  |  |  |  |  |  |
| **Protein modification** | | |  |  |  |
| orf19.1702 | *ARF3* | *ARF3* | 7.7 | 0.0185 | Putative ADP-ribosylation factor 6 |
| orf19.5964 | *ARF2* | *ARF2* | 4.1 | 0.0130 | Putative ADP-ribosylation factor |
|  |  |  |  |  |  |
| **Cell polarity/actin cytoskeleton** | | |  |  |  |
| orf19.6573 | *BEM2* | *BEM2* | 7.0 | 0.0461 | Ras guanine nucleotide exchange factor |
| orf19.3013 | *CDC12* | *CDC12* | 4.7 | 0.0118 | Septin |
| orf19.4413 | *CMD1* | *CMD1* | 4.0 | 0.0297 | Calmodulin |
| orf19.6146 | *CLG1* | *CLG1* | 3.5 | 0.0251 | Predicted cyclin-like protein |
| orf19.390 | *CDC42* | *CDC42* | 3.4 | 0.0303 | Rho-type GTPase |
| orf19.5076 | *PFY1* | *PFY1* | 3.3 | 0.0164 | Profilin |
| orf19.5015 | *MYO2* | *MYO2* | 2.3 | 0.0114 | Putative class V myosin I |
|  |  |  |  |  |  |
| **Other functions** | |  |  |  |  |
| orf19.3981 | *MAL31* | *MAL31* | 7.7 | 0.0161 | Maltose permease |
| orf19.7308 | *TUB1* | *TUB1* | 4.5 | 0.0110 | Alpha-tubulin |
| orf19.4456 | *GAP4* | *GAP1* | 4.3 | 0.0165 | Putative amino acid permease |
| orf19.3668 | *HGT2* | *RGT2* | 3.5 | 0.0071 | Similar to *C. albicans* Hgt1p |
| orf19.5537 | *WSC2* | *WSC2* | 3.4 | 0.0171 | Similar to *S. cerevisiae* Wsc2p |
| orf19.3839 | *SAP10* | *MKC7* | 3.2 | 0.0457 | Secreted aspartyl proteinase |
| orf19.1480 | *SDH6* | *YMR118C* | 3.2 | 0.0036 | Succinate dehydrogenase |
| orf19.4716 | *GDH3* | *GDH3* | 24.4 | 0.0111 | Putative NADP-glutamate dehydrogenase |
|  |  |  |  |  |  |
| **Unknown function** | |  |  |  |  |
| orf19.3710 | *YHB5* |  | 10.9 | 0.0044 | Protein related to flavohemoglobins |
| orf19.6021 | *IHD2* |  | 10.7 | 0.0058 | Unknown function |
| orf19.2432 | *HAC1* |  | 7.0 | 0.0190 | Unknown function |
| orf19.3352 | *-* |  | 6.7 | 0.0461 | Unknown function |
| orf19.1301 | *-* |  | 6.6 | 0.0001 | Protein of unknown function |
| orf19.4666 | *-* |  | 6.0 | 0.0102 | Unknown function |
| orf19.4792 | *-* |  | 5.9 | 0.0074 | Unknown function |
| orf19.699 | *-* |  | 4.9 | 0.0451 | Protein of unknown function |
| orf19.7531 | *-* |  | 4.7 | 0.0343 | Protein of unknown function |
| orf19.2923 | *-* |  | 4.7 | 0.0472 | Putative transporter |
| orf19.3219 | *-* |  | 4.4 | 0.0325 | Unknown function |
| orf19.5495 | *-* |  | 4.4 | 0.0262 | Unknown function |
| orf19.3826 | *-* |  | 4.3 | 0.0219 | Unknown function |
| orf19.7330 | *-* |  | 4.2 | 0.0232 | Unknown function |
| orf19.815 | *-* |  | 4.1 | 0.0118 | Predicted GEF |
| orf19.2769 | *-* |  | 4.0 | 0.0238 | Unknown function |
| orf19.4607 | *-* |  | 4.0 | 0.0372 | Unknown function |
| orf19.7314 | *CDG1* |  | 3.9 | 0.0434 | Similar to cysteine dioxygenases |
| orf19.6318 | *-* |  | 3.7 | 0.0423 | Membrane protein of unknown function |
| orf19.4897 | *SFH5* |  | 3.7 | 0.0401 | Putative phosphatidylinositol transfer protein |
| orf19.3737 | *-* |  | 3.7 | 0.0186 | Unknown function |
| orf19.7579 | *FGR34* |  | 3.6 | 0.0381 | Unknown function |
| orf19.5619 | *-* |  | 3.6 | 0.0058 | Unknown function |
| orf19.6731 | *-* |  | 3.5 | 0.0170 | Unknown function |
| orf19.3843 | *-* |  | 3.4 | 0.0130 | Unknown function |
| orf19.3944 | *-* |  | 3.3 | 0.0141 | Unknown function |
| orf19.5642 | *-* |  | 3.2 | 0.0423 | Unknown function |
| orf19.4914 | *-* |  | 3.2 | 0.0239 | Unknown function |
| orf19.6023 | *ERC2* |  | 3.2 | 0.0004 | Predicted membrane protein |
| orf19.3633 | *-* |  | 3.2 | 0.0239 | Unknown function |
| orf19.2965 | *-* |  | 3.2 | 0.0135 | Unknown function |
| orf19.7060 | *-* |  | 3.1 | 0.0030 | Unknown function |
| orf19.7504 | *-* |  | 3.1 | 0.0275 | Protein of unknown function |
| orf19.7163 | *-* |  | 3.1 | 0.0037 | Unknown function |
| orf19.6608 | *-* |  | 3.1 | 0.0190 | Unknown function |
| orf19.7038 | *-* |  | 3.0 | 0.0140 | Unknown function |
