# Supplementary material for: The Protein Kinase Tor1 Regulates Adhesin Gene Expression in Candida albicans
Source: PLoS Pathog. 2009 Feb 6;5(2):e1000294. doi: 10.1371/journal.ppat.1000294 (PMC2631134; doi:10.1371/journal.ppat.1000294)
Supplement: Table S5 — Strains used in this study (0.10 MB DOC) [file ppat.1000294.s006.doc]

**Table S5**. Strains used in this study

| **Strain** | **Genotype** | **Reference** |
| --- | --- | --- |
| SC5314 | Wild type | [1] |
| JRB12 | SC5314 *TOR1-1/TOR1* | [2] |
| CAYC2 | *ura3Δ::imm434/ura3Δ::imm434 als1Δ::hisG/als1Δ::hisG-URA3-hisG* | [3] |
| CAYF178U | *ura3Δ::λimm434::URA3-IRO1/ura3Δ::λimm434 als3::ARG4/als3::HIS1 arg4::hisG/arg4::hisG his1::hisG/his1::hisG* | [4] |
| CAL4 | *hwp1Δ::hisG/hwp1Δ::hisG ura3Δ::imm434/ura3Δ::imm434* | [5] |
| FJS6 | *ura3Δ::λimm434/ura3Δ::λimm434 arg4::hisG/arg4::hisG his1::hisG/his1::hisG ece1::Tn7-UAU1/ece1::Tn7-URA3* | [4] |
| YAG171 | *ura3Δ::imm434/ura3Δ::imm434 rbp1::MX3/rbp1::CaURA3MX3R* | [2] |
| CJN1348 | *ura3Δ::λimm434::URA3-IRO1/ura3Δ::λimm434 als1::hisG/als1::hisG als3::dpl::200/als3::dpl200* | [6] |
| CJN1352 | *ura3Δ::λimm434::URA3-IRO1/ura3Δ::λimm434 als1::hisG/als1::hisG als3::dpl::200/als3::dpl200 ALS1::pAgTEF1-NAT1-AgTEF1UTR-TDH3-ALS1/ALS1* | [6] |
| CJN1356 | *ura3Δ::λimm434::URA3-IRO1/ura3Δ::λimm434 als1::hisG/als1::hisG als3::dpl::200/als3::dpl200 ALS3::pAgTEF1-NAT1-AgTEF1UTR-TDH3-ALS3/ALS3* | [6] |
| JKC18 | *ura3Δ::λimm434/ura3Δ::λimm434 cph1::hisG/cph1::hisG* | [7] |
| HLY1921 | *ura3Δ::λimm434/ura3Δ::λimm434 his1::hisG/his1::hisG arg4::hisG/arg4::hisG cph2::ARG4/cph2::URA3* | [8] |
| HLC67 | *ura3Δ::λimm434/ura3Δ::λimm434 efg1::hisG/efg1::hisG* | [9] |
| HLC74 | *ura3Δ::λimm434/ura3Δ::λimm434 efg1::hisG/efg1::hisG* (*EFG1*) | [9] |
| CJN896 | *ura3Δ::λimm434/ura3Δ::λimm434 arg4::hisG/arg4::hisG his1::hisG::pHIS1/his1::hisG tec1::Tn7-UAU1/tec1::Tn7-URA3* | [10] |
| CJN702 | *ura3Δ::λimm434/ura3Δ::λimm434 arg4::hisG/arg4::hisG his1::hisG::pHIS1/his1::hisG bcr1::ARG4/bcr1::URA3* | [10] |
| CJN698 | *ura3Δ::λimm434/ura3Δ::λimm434 arg4::hisG/arg4::hisG his1::hisG::pHIS1-BCR1/his1::hisG bcr1::ARG4/bcr1::URA3* | [10] |
| CJN517 | *ura3Δ::λimm434/ura3Δ::λimm434 arg4::hisG/arg4::hisG his1::hisG::pHIS1/his1::hisG czf1::Tn7-UAU1/czf1::Tn7-URA3* | [10] |
| CJN267 | *ura3Δ::λimm434/ura3Δ::λimm434 arg4::hisG/arg4::hisG his1::hisG::pHIS1/his1::hisG rim101::Tn7-UAU1/rim101::Tn7-URA3* | [10] |
| BCa23-3 | *ura3Δ::λimm434/ura3Δ::λimm434 nrg1::hisG/nrg1::hisG* | [11] |
| BCa23-7 | *ura3Δ::λimm434/ura3Δ::λimm434 nrg1::hisG/nrg1::hisG ACT1::NRG1/ACT1* | [11] |
| BCa2-9 | *ura3Δ::λimm434/ura3Δ::λimm434 tup1::hisG/tup1::hisG* | [12] |
| BCa2-11 | *ura3Δ::λimm434/ura3Δ::λimm434 tup1::hisG/tup1::hisG* (*TUP1*) | [12] |
| DK128 | *ura3Δ::λimm434/ura3Δ::λimm434 rfg1::hisG/rfg1::hisG-URA3-hisG* | [13] |
| ATCC 6260 | *Candida guilliermondii* (Castellani) |  |

**References**

1. Fonzi WA, Irwin MY (1993) Isogenic strain construction and gene mapping in *Candida albicans*. Genetics 134: 717-728.

2. Cruz MC, Goldstein AL, Blankenship J, Del Poeta M, Perfect JR, et al. (2001) Rapamycin and less immunosuppressive analogs are toxic to *Candida albicans* and *Cryptococcus neoformans* via FKBP12-dependent inhibition of TOR. Antimicrob Agents Chemother 45: 3162-3170.

3. Fu Y, Ibrahim AS, Sheppard DC, Chen YC, French SW, et al. (2002) *Candida albicans* Als1p: an adhesin that is a downstream effector of the *EFG1* filamentation pathway. Mol Microbiol 44: 61-72.

4. Nobile CJ, Andes DR, Nett JE, Smith FJ, Yue F, et al. (2006) Critical role of Bcr1-dependent adhesins in *C. albicans* biofilm formation in vitro and in vivo. PLoS Pathog 2: e63.

5. Sharkey LL, McNemar MD, Saporito-Irwin SM, Sypherd PS, Fonzi WA (1999) *HWP1* functions in the morphological development of *Candida albicans* downstream of *EFG1*, *TUP1*, and *RBF1*. J Bacteriol 181: 5273-5279.

6. Nobile CJ, Schneider HA, Nett JE, Sheppard DC, Filler SG, et al. (2008) Complementary adhesin function in *C. albicans* biofilm formation. Curr Biol 18: 1017-1024.

7. Liu H, Kohler J, Fink GR (1994) Suppression of hyphal formation in *Candida albicans* by mutation of a *STE12* homolog. Science 266: 1723-1726.

8. Lane S, Zhou S, Pan T, Dai Q, Liu H (2001) The basic helix-loop-helix transcription factor Cph2 regulates hyphal development in Candida albicans partly via *TEC1*. Mol Cell Biol 21: 6418-6428.

9. Lo HJ, Kohler JR, DiDomenico B, Loebenberg D, Cacciapuoti A, et al. (1997) Nonfilamentous *C. albicans* mutants are avirulent. Cell 90: 939-949.

10. Nobile CJ, Mitchell AP (2005) Regulation of cell-surface genes and biofilm formation by the *C. albicans* transcription factor Bcr1p. Curr Biol 15: 1150-1155.

11. Braun BR, Kadosh D, Johnson AD (2001) *NRG1*, a repressor of filamentous growth in *C.albicans*, is down-regulated during filament induction. EMBO J 20: 4753-4761.

12. Braun BR, Johnson AD (1997) Control of filament formation in *Candida albicans* by the transcriptional repressor *TUP1*. Science 277: 105-109.

13. Kadosh D, Johnson AD (2001) Rfg1, a protein related to the *Saccharomyces cerevisiae* hypoxic regulator Rox1, controls filamentous growth and virulence in *Candida albicans*. Mol Cell Biol 21: 2496-2505.
