# Supplementary material for: The Protein Kinase Tor1 Regulates Adhesin Gene Expression in Candida albicans
Source: PLoS Pathog. 2009 Feb 6;5(2):e1000294. doi: 10.1371/journal.ppat.1000294 (PMC2631134; doi:10.1371/journal.ppat.1000294)
Supplement: Table S6 — Primers used for northern probe amplification (0.06 MB DOC) [file ppat.1000294.s007.doc]

**Table S6.** Primers used for northern probe amplification

| **Gene probe** | **Primer name** | **Primer sequence** | **Probe size** |
| --- | --- | --- | --- |
| *ALS1* Sense | JOHE18703 | 5'-CAACTACATTATCAACTACTTCAAACTC-3' | 500 bp |
| *ALS1* Antisense | JOHE18704 | 5'-AGTAACAGATCCACTAGTAACAAGTTC-3' |  |
| *ALS3* Sense | JOHE18709 | 5'-GTTGAAACAGAATCTTCTACTGTTACTACT-3' | 523 bp |
| *ALS3* Antisense | JOHE18710 | 5'-TAGTAAGCATATGTAAACTAACTAAAGATG-3' | |
| *HWP1* Sense | JOHE17704 | 5'-TTCTTCTTCATCATCATCTACTACTC-3' | 397 bp |
| *HWP1* Antisense | JOHE17705 | 5'-GTAAGTGGTGTAAATAGTATCTTTAGATGT-3' | |
| *ECE1* Sense | JOHE18533 | 5'-CTACTGTTTTTGCTTTATCTTCTCA-3' | 700 bp |
| *ECE1* Antisense | JOHE18534 | 5'-CTCTTTTTGATTAAATTGCTAAGTG-3' |  |
| *NRG1* Sense | JOHE17710 | 5'-CATACCATCAACAATACTACAACTATC-3' | 337 bp |
| *NRG1* Antisense | JOHE17711 | 5'-CTAGCTAAATGTCCTGAAGTAGTAAAC-3' |  |
| *TUP1* Sense | JOHE17712 | 5'-AGAAGATTATGACTCAAAGTACCAAC-3' | 313 bp |
| *TUP1* Antisense | JOHE17713 | 5'-AATGTATTGTGACTTGTCGATAACC-3' |  |
| *ACT1* Sense | JOHE17730 | 5'-AGAGACATTAAAGAAAGATTGTGTTAC-3' | 363 bp |
| *ACT1* Antisense | JOHE17731 | 5'-AATAATCTTAACTTTCATAGAAGATGG-3' |  |
